# Structural and Behavioral Correlates of HIV Infection among Pregnant Women in a Country with a Highly Generalized HIV Epidemic: A Cross-Sectional Study with a Probability Sample of Antenatal Care Facilities in Swaziland

**DOI:** 10.1371/journal.pone.0168140

**Published:** 2016-12-12

**Authors:** Bhekumusa Wellington Lukhele, Teeranee Techasrivichien, S. Pilar Suguimoto, Patou Masika Musumari, Christina El-saaidi, Samson Haumba, Oslinah Buru Tagutanazvo, Masako Ono-Kihara, Masahiro Kihara

**Affiliations:** 1 Department of Global Health and Socio-epidemiology, School of Public Health, Graduate School of Medicine, Kyoto University, Kyoto, Japan; 2 Center for Medical Education, Graduate School of Medicine, Kyoto University, Kyoto, Japan; 3 Center for Human Services, University Research Co., LLC, Mbabane, Swaziland; 4 Department of Midwifery Science, Faculty of Health Sciences, University of Swaziland, Mbabane, Swaziland; Leibniz Institute for Prvention Research and Epidemiology BIPS, GERMANY

## Abstract

**Introduction:**

HIV disproportionately affects women in Sub-Saharan Africa. Swaziland bears the highest HIV prevalence of 41% among pregnant women in this region. This heightened HIV-epidemic reflects the importance of context-specific interventions. Apart from routine HIV surveillance, studies that examine structural and behavioral factors associated with HIV infection among women may facilitate the revitalization of existing programs and provide insights to inform context-specific HIV prevention interventions.

**Methods and Findings:**

This cross-sectional study employed a two-stage random cluster sampling in ten antenatal health care facilities in the Hhohho region of Swaziland in August and September 2015. Participants were eligible for the study if they were 18 years or older and had tested for HIV. Self-administered tablet-based questionnaires were used to assess HIV risk factors. Of all eligible pregnant women, 827 (92.4%) participated, out of which 297 (35.9%) were self-reportedly HIV positive. Among structural factors, family function was not significantly associated with self-reported HIV positive status, while lower than high school educational attainment (AOR, 1.65; CI, 1.14–3.38; *P* = 0.008), and income below minimum wage (AOR, 1.81; CI, 1.09–3.01; *P* = 0.021) were significantly associated with self-reported HIV positive status. Behavioral factors significantly associated with reporting a positive HIV status included; ≥2 lifetime sexual partners (AOR, 3.16; CI, 2.00–5.00; *P*<0.001), and ever cohabited (AOR, 2.39; CI, 1.66–3.43; P = 0.00). The most cited reason for having multiple sexual partners was financial gain. HIV/AIDS-related knowledge level was high but not associated to self-reported HIV status (P = 0.319).

**Conclusions:**

Structural and behavioral factors showed significant association with self-reported HIV infection among pregnant women in Swaziland while HIV/AIDS-related knowledge and family function did not. This suggests that HIV interventions should be reinforced taking into consideration these findings. The findings also suggest the importance of future research sensitive to the Swazi and African sociocultural contexts, especially research for family function.

## Introduction

According to the World Health Organization, HIV/AIDS-related death is the worldwide leading cause of death among women of reproductive age [1]. Globally, 15% of women living with HIV in 2013 were of age 15–24 years, of whom 80% lived in Sub-Saharan Africa (SSA) [2]. Within the SSA region, the burden of HIV among women of age 15–49 years varies considerably; 7.6% in Kenya (2014) [3], 16.9% in Namibia (2014) [4], 19.0% in South Africa (2015) [5] and Swaziland bears the highest HIV prevalence of 38.8% (2011) [6]. The majority (62%) of all new infections in Swaziland occur among women[7]. HIV prevalence in Swaziland is higher among women aged 18–49 years (38.8%) compared to their male counterparts of the same age group (23.1%), and particularly high among women aged 30–34 years old, 54% compared to 37% in men of the similar age group [6]. Although there have been reports of a decline in HIV prevalence among women in southern Africa [8,9], at best, the epidemic in Swaziland seems to have only stabilized [6,10].

### Young women’s vulnerability to HIV

Young women’s vulnerability to HIV could be attributed to several factors. First, families have great influence among young women. Studies show that lack of parental monitoring, poor parent-child communication, and low cohesion among family members are associated with increased HIV risky sexual behavior [11–16]. Second, research has shown a strong link between HIV/AIDS and poverty. Frequently, poverty drives girls and women to exchange sex for food or basic amenities and cause a day-to-day existence dominated by immediate survival needs and indifference to high HIV risk sexual behaviors [17–21]. Furthermore, most young women growing up in economically deprived families have little access to schooling and few future prospects, therefore, find themselves coerced into sexual activity with older working men for survival [22,23]. Third, behavioral factors such as; early sexual debut [24–27], inconsistent condom use [28–30], multiple sexual partnerships (concurrent and serial) [31–33], poor sexual-decision making under the influence of alcohol [34–36] and others, have shown to increase HIV vulnerability among young women. Lastly, there is extensive literature identifying biological factors putting women at particularly higher risk of HIV compared to men. As pointed out by Ramjee and colleague “women have a greater vaginal mucosal surface area exposed to pathogens and infectious fluid for longer periods during sexual intercourse, and that young women are particularly at higher risk due to cervical ectopy which facilitates greater exposure of target cells to trauma and pathogenesis in the vagina [9]”.

### Review of previous studies in Swaziland

In Swaziland, there are limited studies specifically focusing on HIV vulnerability among young women. Existing research has focused on men who have sex with men [37], sex workers [38], in-school youth [39] and the general population [40,41]. Studies on young women have either been qualitative [42–44] -which are suited to exploring risks at in-depth levels, but, fall short in quantifying risk- or sentinel surveillance studies using face-to-face interviews [45] which afford less privacy and anonymity and thus likely increase motivational bias [46,47]. This study aims to investigate HIV risk factors between HIV negative and HIV positive young women to provide empirical evidence specific to Swaziland. To achieve this goal, we studied pregnant women attending antenatal care since the median age at first birth is 19.8 years [48] and the majority (98.5%) of pregnant women access antenatal care services in Swaziland [49]. We used simple two-stage cluster sampling and self-administered computer-assisted data collection technique to overcome shortcomings of prior research.

## Methods

### Ethical considerations

This study was conducted according to the ethical principles outlined in the Declaration of Helsinki. The research protocol was approved by the Kyoto University Faculty of Medicine and Graduate School of Medicine, Ethics Committee, Japan (R0073) and the Swaziland Scientific and Ethics Committee, Swaziland (MH/599C/FWA00015267/IRB0009688). All participants signed a written informed consent. One USD (1$) was offered to each participant as compensation for taking part in our study.

### Study setting

Swaziland, is a small land-locked country situated in Southern Africa. Its area is approximately 17 364 km^2^ with an estimated population of 1 287 050 (2015), of which about 76% reside in rural areas [50]. Swaziland is divided into four administrative regions: Hhohho, Manzini, Lubombo, and Shiselweni region. The number of health facilities that provide antenatal care services to pregnant women per region is as follows: 52 in Hhohho, 63 in Manzini, 38 in Lubombo and 30 in Shiselweni [50]. In the Hhohho region, of all the facilities which offer antenatal health services, 78.8% are public and the rest (21.2%) are private facilities. The Hhohho region was selected as our study setting since it has the highest generalized HIV prevalence in the country; 27.8% in Hhohho, 21.9% in Shiselweni, 20.7% in Manzini, and 20.5% in Lubombo [40].

### Participants

Our study targeted pregnant women who were ≥18 years old, had tested for HIV and were attending antenatal care services at facilities in the Hhohho region for the first time during the study period. We calculated the sample size following the approach proposed by Kohn et al and Hulley at al [51,52]. We based the calculation on results from the sentinel surveillance report which showed that 41% were HIV positive and 59% were HIV negative [45], to detect the difference in parental monitoring proportion of 28% and 52% [53] among HIV positive and negative participants respectively at *α* = 0.05, *β* = 0.2. Based on these, a total sample size of 149 (for both groups) was sufficient to detect this difference. Taking into consideration the complex sample design effect of 2.0 [54], we inflated the sample size by a factor of two, resulting in a sample size of 298.

We further increased the sample size to 596 by multiplying by a factor of two to ensure the statistical power is enough for multivariate analysis. Finally, the sample size was adjusted to 894, assuming a response rate of two-thirds due to the sensitive nature of our questionnaire.

### Survey instrument

A self-administered structured questionnaire was developed in English based on the review of Swazi and international literature [47]. To improve the initial draft [47], we conducted a preliminary qualitative study during February—March 2015 using semi-structured in-depth interviews among 37 pregnant women recruited through purposive sampling. We recruited pregnant women in their 3^rd^ trimester to ensure that they would not be re-sampled for the current subsequent quantitative study. This initial step served several aims [55]. First, it allowed us to explore in-depth the sexual histories and ease of recalling those histories. Second, it enabled us to resolve language discrepancies to improve the translated draft. Lastly, it provided insights into recruitment issues. As described in our previous work, “the modified draft was then converted into an electronic format compatible with internet-enabled tablets, designed to be user-friendly and intuitive even for those participants not familiar with electronic devices” [47]. Using the tablet-based questionnaire, we piloted the instrument among 14 pregnant women (from a health facility not included in our survey sites) to test for face validity, skip logic, user interface, time to complete the survey and the upload-download functionality of the software.

The final survey instrument (S1 Questionnaire) consisted of a question on HIV status and seven domains: sociodemographic characteristics (6 items), schooling characteristics (2 items), HIV/AIDS-related knowledge (8 items), childhood household ownership of durable assets (19 items), obstetric characteristic (1 item), family characteristics (33 items) and sexual history characteristics (13 items). The family characteristics domain contained three items about parental characteristics and three subscales about family function: a) family cohesion subscale (8 items, Cronbach’s alpha = 0.63), b) parental monitoring subscale (6 items, Cronbach’s alpha = 0.67), and c) parent-child communication subscale (16 items, Cronbach’s alpha = 0.83). The domain of sexual history characteristics explored the current and past sexual behavior. Items on HIV/AIDS-related knowledge and sexual histories were in part taken from the Swaziland Demographic Health Survey [40]. In the absence of a locally validated family function scale, we adapted items from Family Adaptability and Cohesion Evaluation Scales IV (FACES IV) [56] as well as the Parent Monitoring Scale [53]. The instrument was translated into the local language (siSwati) by the bilingual researcher BWL and back-translated by another independent researcher to minimize translation dissonance.

### Study design and sampling

The survey was a cross-sectional study using a simple two-stage cluster sampling strategy following Levy and Lemeshow [57]. The National Monitoring and Evaluation Office at the Ministry of Health in Swaziland facilitated us with the list of all 52 health facilities providing antenatal care services in the Hhohho region. Each facility was considered as a cluster in our study. In the first stage, we selected 10 clusters using simple random sampling without replacement. In the second stage, we enumerated 41 working days during August and September 2015, excluding Swazi Holidays and weekends, to serve as listing units. Then, we selected one working day to serve as a start date for the survey using simple random sampling. We estimated that twenty working days were sufficient to cover our desired sample size and prevent bias due to variations in weekly cycles. All pregnant women presenting at the 10 health facilities (10 clusters) from the random start date (17 August 2015) were consecutively screened for eligibility and invited to participate in the study. Recruitment took place throughout working hours in all facilities.

### Data collection

To ensure high-quality data collection, we recruited nurses as field staff and provided them with a two-day intensive training; one day at a central location and another day at the data collection site. The field staff was trained on ethical considerations, aims and objectives of the study, the tablet use, and how to integrate the survey within patient flow. We followed a similar protocol for our previous research [47], having our field staff carry the print outs of screenshots of the electronic questionnaire to be able to read out loud and guide participants who had proficiency challenges without the field staff having to see their responses. BWL supervised data collection.

### Statistical analysis

#### Sample weights and design effect

All statistical analyses were carried out using Complex Sample module of SPSS version 21 to account for the two-stage cluster sampling. We considered our sample self-weighted because, even though the selection of antenatal care clusters was done through simple random sampling at the first stage, at the second stage, pregnant women were consecutively sampled from all walk-in eligible potential participants, ensuring the sample size was potentially proportional to the total number of pregnant women attending each facility [54]. We calculated point estimates (proportions), their standard errors (SEs), and 95% confidence intervals (CIs) accounting for cluster sample design [57,58]. The magnitude of the inflation in variance was measured as the design effect, defined as “the ratio of the actual variance of a sample to the variance of a simple random sample of the same number of elements” [59].

#### Childhood household wealth index

Childhood household wealth index was developed according to the procedure described by Vyas and Kumaranayake [60]. Briefly, participants were asked if their childhood household had any of the 19 durable assets listed in the questionnaire (refer to S1 Questionnaire). Having the asset was coded as “1” and not having the asset as “0”. The data was then analyzed using principal component analysis (PCA) which revealed that the first component included 10 items and accounted for 25.5% of all variance. Childhood household wealth index was defined as the total score of these 10 items weighted with the factor load of each item. After that, we ranked the participants into quintiles from poorest to the wealthiest according to their total score [61].

#### Family function

Family function consisted of three subscales (refer to S1 Questionnaire), to measure family cohesion, parental monitoring and parent-child communication, as previously stated. All responses of these subscales were 5-point Likert scale from “strongly disagree” to “strongly agree”. In the analysis, responses were coded in the same direction such that higher scores represented “better family function” on all responses. For each subscale, we calculated the composite score, which was further divided into quintiles ranging from the lowest to the highest.

#### HIV/AIDS-related knowledge

HIV/AIDS-related knowledge included eight questions (refer to S1 Questionnaire). The total score was summed (min 0—max 8) (S1 Table) and later categorized as either ‘high” (correct response ≥7) or “low” (correct response ≤6).

#### Bivariate and multiple logistic regressions

Bivariate analysis was performed using Chi-square tests for categorical variables to determine associations between HIV status and other variables. Factors that were significantly associated with being HIV positive at *P* value ≤ 0.10 were considered candidates to be included in the multiple logistic regression analysis. To provide a better fit for our multiple logistic regression model, we polychotomized continuous variables since their distributions were nonlinear. Out of 21 factors associated with HIV status at *P* value ≤ 0.10 in the bivariate analysis, 7 were excluded based on epidemiological importance or because they were subset questions of upstream questions like “currently in a polygamous marriage” a subset question for those who reported being married. There was no evidence of multicollinearity and singularity among the remaining factors. All 14 factors were compulsorily entered into the multivariate model to calculate the adjusted odds ratios (AORs) to assess the magnitude of independent association of these predictors with a self-reported HIV positive status.

## Results

Of 894 eligible pregnant women invited to participate, 827 participants completed the study (response rate of 92.5%). The median age was 25 years; the youngest respondent was 18 years old and the oldest 43 years old. Table 1 displays the characteristics of respondents. About half of the respondents had completed at least secondary school (51.3%) and had ever dropped out of school (54.7%). Only 14.6% had ever stayed at a boarding school. The majority (84.9%) lived below Swaziland’s monthly minimum wage (approximately $110 USD), did not have formal employment (58.2%), and were never married (58.5%). Most participants identified correct responses to HIV/AIDS- related knowledge questions, correct responses ranged from 83.1% to 96.0% (S1 Table).

**Table 1:** Descriptive and bivariate factors associated with HIV infection.

		Total N = 827	% of total	HIV positive	% HIV positive	Complex SE	DEFF	P value
**Demographic Variables**								
Age groups								
	18–24	391	47.3	95	24.3	2.5	1.59	<0.001
	25–34	356	43.0	171	43.0	3.6	2.31	
	35–43	80	9.7	31	38.8	3.9	0.64	
Marital status								
	Single	416	50.3	147	35.3	2.7	1.62	0.794
	Married	325	39.3	116	35.7	3.2	1.80	
	Cohabiting	68	8.2	26	38.2	8.5	2.60	
	Ever been married(Divorced and separated)	18	2.2	8	44.4	11.4	0.76	
Level of education								
	Low (<High School)	422	51.0	184	43.6	2.1	0.94	0.001
	High (≥High School)	405	49.0	113	27.9	2.4	1.34	
Employment status								
	Employed	198	23.9	72	36.4	4.3	1.93	0.052
	Not employed	481	58.2	173	36.0	2.1	1.18	
	Student	63	7.6	13	20.6	5.3	1.36	
	Self employed	85	10.3	39	45.9	5.4	1.26	
Level of income								
	≤Minimum wage	702	84.9	265	37.8	1.9	1.35	0.039
	>Minimum wage	125	15.1	32	25.6	4.7	1.76	
Religious services attendance								
	At least once a week	724	87.5	271	37.4	2.3	2.07	0.165
	At least once a month	48	5.8	12	25.0	7.5	1.79	
	At least once a year	17	2.1	6	35.3	9.4	0.81	
	Less than once a year	13	1.6	3	23.1	9.7	0.85	
	Never	25	3.0	5	20.0	6.6	0.84	
**Schooling characteristics**								
Boarding school^a^								
	Yes	61	7.4	9	14.8	4.4	1.18	0.005
	No	740	89.5	275	37.2	1.9	1.36	
Ever dropped out of school								
	Yes	452	54.7	199	44.0	1.9	0.82	<0.001
	No	375	45.3	98	26.1	2.0	0.96	
**Childhood household wealth index**								
Childhood household wealth index								
	Lower wealth (≤Medium)	496	60.0	207	41.7	2.4	1.41	<0.001
	Higher wealth (>Medium)	331	40.0	90	27.2	2.5	1.27	
**Obstetric characteristic**								
Planned pregnancy								
	Yes	312	37.7	110	35.3	3.5	2.12	0.817
	No	515	62.3	187	36.3	2.6	1.93	
**Family characteristics**								
**Family function**								
Family Cohesion								
	Lowest	178	21.5	70	39.4	6.1	3.39	0.750
	Low	149	18.0	53	35.6	2.8	0.61	
	Medium	160	19.3	55	34.4	1.7	0.25	
	High	164	19.8	56	34.2	3.6	1.17	
	Highest	176	21.3	63	35.8	4.8	2.21	
Parental Monitoring								
	Lowest	150	18.1	55	36.7	4.0	1.26	0.532
	Low	212	25.6	83	39.2	2.4	0.64	
	Medium	144	17.4	52	36.1	2.8	0.61	
	High	182	22.0	64	35.2	4.0	1.59	
	Highest	139	16.8	43	30.9	5.3	2.22	
Parent-Child Communication								
	Lowest	165	20.0	58	35.2	5.7	2.86	0.759
	Low	171	20.7	62	36.3	3.1	0.87	
	Medium	177	21.4	58	32.8	4.1	1.67	
	High	158	19.1	61	38.6	3.4	0.98	
	Highest	156	18.9	58	37.2	5.0	2.03	
**Parental Characteristics**								
Father had polygamy								
	Yes	272	32.9	108	39.7	3.9	2.15	0.281
	No/ don’t know	555	67.1	189	34.1	2.7	2.18	
Parents had multiple sexual partners								
	Yes	301	36.4	113	37.5	3.3	1.76	0.434
	No/ don’t know	526	63.6	184	35.0	2.1	1.22	
HIV related death of a family member								
	Yes	430	52.0	171	40.0	2.2	1.062	0.001
	No	397	48.0	126	31.7	2.4	1.269	
**Sexual History**								
Currently in a polygamous union^b^								
	Yes	27	3.3	14	51.9	9.8	1.28	0.041
	No	298	36.0	102	34.2	2.7	1.19	
Lifetime number of sexual partners								
	1	208	25.2	31	14.9	1.8	0.68	<0.001
	≥2	619	74.8	266	43.0	2.6	2.05	
Multiple sexual partners in the past 12 months								
	1	709	85.7	239	33.7	1.9	1.36	0.001
	≥2	118	14.3	58	49.2	4.2	1.03	
Perceived reason for multiple sexual partnerships								
	Lust	130	15.7	46	35.4	4.7	1.55	0.007
	Financial benefit	394	47.6	143	36.3	2.3	1.10	
	Fear of disappointment from current partner	95	11.5	45	47.4	5.4	1.34	
	Sexually unsatisfied with current partner	40	4.8	13	32.5	3.5	0.27	
	Looking for adventure	30	3.6	8	26.7	7.2	0.98	
	Peer Pressure	25	3.0	3	12.0	3.3	0.33	
	Lack of knowledge of risks of HIV	78	9.4	30	38.5	3.4	0.48	
	Get tempted to have sex	27	3.3	4	14.8	4.1	0.45	
	Other	8	1.0	5	62.5	9.6	0.39	
Condom use at last sex								
	Yes	336	40.6	161	47.9	3.7	2.25	0.001
	No	491	59.4	136	27.7	2.4	1.70	
Condom use at first sex								
	Yes	401	48.5	111	27.7	2.1	1.13	0.004
	No	426	51.5	186	43.7	3.6	2.74	
Age at sexual debut								
	≤17	327	39.5	132	40.4	3.4	1.91	0.025
	>17	500	60.5	165	33.0	1.6	0.71	
Intergenerational sex at sexual debut								
	>10 years older	120	14.5	54	45.0	3.4	0.69	0.026
	<10 years older	707	85.5	243	34.4	2.3	2.05	
Ever cohabited								
	Yes	228	27.6	121	53.1	3.2	1.20	<0.001
	No	599	72.4	176	29.4	2.0	1.44	
Knew first sexual partner's HIV status								
	Yes	211	25.5	43	20.4	4.2	2.55	0.005
	No	616	74.5	254	41.2	2.5	1.77	
Know current sexual partner’s HIV status								
	Yes	598	72.3	195	32.6	2.3	1.77	0.042
	No	229	27.7	102	44.5	4.6	2.45	
Ever experienced forced sex								
	Yes	251	30.4	106	42.2	2.5	0.81	0.006
	No	576	69.6	191	33.2	2.4	1.91	
Ever had sex under the influence of alcohol								
	Yes	110	13.3	47	42.7	5.4	1.63	0.107
	No	717	86.7	250	34.9	1.9	1.45	
HIV/AIDS related knowledge level								
	High (≥7)	650	78.6	238	36.6	2.6	2.28	0.319
	Low (≤6)	177	21.4	59	33.3	2.1	0.48	

Complex SE = Standard error of estimate under complex sampling analysis.

DEFF = Design effect.

^b^ = “currently in a polygamous union” was asked only among those who were married (n = 325).

^a^ = “boarding school” excluded those who did not complete primary education (n = 801).

P value was calculated using the second-order Rao-Scott adjusted chi-square statistic.

### Prevalence of self-reported HIV status by characteristics of participants

Overall, self-reported HIV prevalence was 35.9%. As displayed in Table 1, those who reported an HIV positive status were more likely to be older (*P*<0.001), have lower than high school educational attainment (*P* = 0.001), have ever dropped out of school (*P*<0.001), be self-employed (*P* = 0.052), lived below Swaziland’s monthly minimum wage (*P* = 0.039), never stayed at a boarding school (*P* = 0.005), have had a lower childhood household wealth index (*P*<0.001), in a polygamous union (*P* = 0.041), had two or more lifetime number of sexual partners (*P*<0.001), had multiple sexual partners (MSP) in the past 12 months (*P* = 0.001), used condom at last (*P* = 0.001) and first sex(*P* = 0.004), had sexual debut at 17 years or younger, experienced intergenerational sex at sexual debut (*P* = 0.025), had ever cohabited (*P*<0.001), did not know their first or current partner’s HIV status (*P* = 0.005 or 0.042), and had ever experienced forced sex (*P* = 0.006). Marital status, religious services attendance, planned pregnancy, parental cohesion, parental monitoring parent-child communication, father with polygamous union or partners having MSP, ever had sex under the influence of alcohol and high HIV/AIDS-related knowledge were not significantly associated with a reported positive HIV status (*P* > 0.05)

### Bivariate associations between independent variables and self-reported HIV status

As shown in Table 2, factors significantly associated with self-reported HIV status included older age 25–34 years [Crude Odds Ratio (COR), 2.88; CI, 1.85–4.48; *P*<0.001] and 35–43 years (COR, 1.97; CI, 1.39–2.79; *P*<0.001) compared to 18–24 years, lower than high school educational attainment (COR, 2.00; CI, 1.47–2.71; *P*<0.001), level of income less than Swaziland’s monthly minimum wage (COR, 1.76; CI,1.03–3.02; *P* = 0.040), lower childhood household wealth index (COR, 1.92; CI,1.45–2.54; *P*<0.001), ≥2 lifetime number of sexual partners (COR, 4.30; CI, 2.97–6.24; *P*<0.001), condom use during last sex (COR, 2.40; CI,1.56–3.70; *P*<0.001), no condom use at first sexual debut (COR, 2.03; CI,1.32–3.10; *P*<0.001), ≤ 17 years old at sexual debut (COR, 1.37; CI,1.11–1.80; *P* = 0.034), intergenerational sex at first sexual debut (COR, 1.56; CI, 1.10–2.29; *P* = 0.030), ever cohabited (lived with a man as if married) (COR, 2.72; CI, 2.00–3.69; *P*<0.001), knew first sexual partner’s HIV status (COR, 2.74; CI,1.46–5.16; *P* = 0.005), know current sexual partner’s HIV status (COR, 1.66; CI, 1.21–2.27; *P* = 0.042), ever experienced forced sex (COR, 1.47;1.15–1.89; *P =* 0.006) and HIV related death of a family member (COR, 1.42; CI,1.20–1.68; *P* = 0.001).

**Table 2:** Factors associated with reported HIV positive status by binary logistic and multiple logistic regression among 827 respondents.

		COR	95% CI^a^	P value	AOR	95%CI	P value
Age groups							
	18–24	Ref			Ref		
	25–34	2.88	1.85–4.48	<0.001	2.38	1.65–3.43	<0.001
	35–43	1.97	1.39–2.79	<0.001	1.31	0.72–2.37	0.380
Level of education							
	Low (<High School)	2.00	1.47–2.71	<0.001	1.65	1.14–3.38	0.008
	High (≥High School)	Ref			Ref		
Level of income							
	< Minimum wage	1.76	1.03–3.02	0.040	1.81	1.09–3.01	0.021
	≥Minimum wage	Ref			Ref		
Childhood household wealth index							
	Lower wealth (≤Medium)	1.92	1.45–2.54	<0.001	1.28	0.88–1.84	0.194
	High wealth (>Medium)	Ref			Ref		
Lifetime number of sexual partners							
	1	Ref			Ref		
	≥2	4.30	2.97–6.24	<0.001	3.16	2.00–5.00	<0.001
Condom use at last sex							
	Yes	2.40	1.56–3.70	<0.001	2.92	2.08–4.10	<0.001
	No	Ref			Ref		
Condom use at first sex							
	Yes	Ref			Ref		
	No	2.03	1.32–3.10	<0.001	1.56	1.10–2.22	0.012
Age at sexual debut							
	≤17	1.37	1.11–1.80	0.034	1.07	0.75–1.53	0.708
	>17	Ref			Ref		
Intergenerational sex at sexual debut							
	>10 years older	1.56	1.10–2.29	0.030	1.43	0.91–2.26	0.126
	<10 Years older	Ref			Ref		
Ever cohabited							
	Yes	2.72	2.00–3.69	<0.001	2.39	1.66–3.43	<0.001
	No	Ref			Ref		
Knew first sexual partner's HIV status							
	Yes	Ref			Ref		
	No	2.74	1.46–5.16	0.005	1.57	1.02–2.42	0.039
Know current sexual partner’s HIV status							
	Yes	Ref			Ref		
	No	1.66	1.21–2.27	0.042	1.47	1.02–2.12	0.038
Ever experienced forced sex							
	Yes	1.47	1.15–1.89	0.006	1.10	0.77–1.58	0.601
	No	Ref			Ref		
HIV related death of a family member							
	Yes	1.42	1.20–1.68	<0.001	1.10	0.78–1.52	0.632
	No	Ref			Ref		

95% CI^a^ = 95% confidence intervals adjusted for cluster sampling in SPSS complex sampling module.

COR = Crude Odds Ratio.

AOR = Adjusted Odds Ratio.

Ref = Reference category.

### Multivariate analysis

As shown in Table 2, factors strongly associated with HIV in the multiple logistic regression analysis included; 25–34 age group [Adjusted Odds Ratio (AOR), 2.38; CI, 1.65–3.43; *P*<0.001], lower than high school educational attainment (AOR, 1.65; CI, 1.14–3.38; *P* = 0.008], and level of income less than Swaziland’s monthly minimum wage (AOR, 1.81; CI, 1.09–3.01, *P* = 0.021). Those who had ≥2 lifetime number of sexual partners were over 3 times more likely to report being HIV positive (AOR, 3.16; CI, 2.00–5.00; *P*<0.001) followed by those who reported condom use during the last sex (AOR, 2.92; CI, 2.08–4.10; *P*<0.001) and no condom use at first sex (AOR, 1.56; CI, 1.10–2.22; *P* = 0.012). Ever cohabited (AOR, 2.39; CI, 1.66–3.43; *P*<0.001), did not know first partner’s HIV status (AOR, 1.57; CI, 1.02–2.42; *P* = 0.039) and does not know current partner’s HIV status (AOR, 1.47; CI, 1.02–2.12; *P* = 0.038) were significantly associated with self-reported HIV infection. We found that childhood household wealth index, sexual debut at ≤ 17 years of age, intergenerational sex (first sexual partner ≥ 10 years older) and HIV-related death of a family member were not significantly associated with HIV infection.

## Discussion

In this study, we explored the association of structural and behavioral factors with self-reported HIV status among pregnant women in Swaziland, a country having the highest generalized HIV epidemic in the world. The high access rate to antenatal care services in Swaziland (98.5%) and high acceptance of HIV testing during antenatal care visits (95.3%) enabled us to assess HIV status without burdening participants with an additional HIV test [49]. Our study revealed that 36% of pregnant women were self-reportedly HIV positive with a peak rate of 52.3% in the age group of 30–34 years. Our findings are corroborated by recent national household survey data which showed that 39% women were HIV positive with a peak of 54% among the age group of 30–34 years, suggesting that our sample is unlikely biased in this respect [6]. We found that family function and HIV/AIDS-related knowledge had no significant statistical association with self-reported HIV infection whereas lower educational attainment, lower income, and certain sexual behaviors were significantly associated with self-reported HIV infection.

### Familial factors

One of our study’s most important findings was that family function (family cohesion, parental monitoring and parent-child communication) was not significantly associated with self-reported HIV status, even after controlling for other factors such as economic status. Though evidence from most published literature shows a significant association between constructs of family function with sexual reproductive outcomes such as sexually transmitted infections [11,12,62–66], we did not find such an association in our study.

There may be several reasons for this. First, family cohesion, parental monitoring, and parent-child communication may not have major influence on HIV infection risk in Swazi’s context where the living arrangement and family structure are mainly of the extended family type [67] with generally higher family function compared to western societies. Western societies predominantly consist of nuclear family types and individualistic life styles [68], and many of the current studies were conducted in these contexts. The second reason may be that our participants were too homogenous in terms of family characteristics to detect such an association. In this case, future studies assessing family characteristics using cluster sampling should consider maximizing heterogeneity among participants by reducing samples within clusters and increasing the number of clusters as suggested by Kish [59]. Third, pregnant women may recall their personal childhood family circumstances and relationship differently, mediated by emotional and psychological changes induced by the current pregnancy. Forth, it is possible that existing family function scales are not sensitive enough to detect Swazi or African specific family function. If this is the case, there is a need for the development of more culturally specific assessment scales to assess family function in future research.

The only familial factor associated with HIV infection was the HIV-related death of a family member. Since participants having a family member who was infected with HIV appeared less likely to have multiple lifetime sexual partners (r = -0.135, *P*<0.001), it is possible that such association is not due to residual effect of statistically unadjusted sexual behavior but may be due to a more frequent HIV testing among participants with such family history.

### Education and financial status

There was a clear inverse dose-response relationship between educational attainment and HIV infection; the higher the education attainment, the lower the reported HIV positive rate (40–50% rate among those with only up to primary or secondary education and 16.8% among those who had tertiary education). The association between education and HIV infection remained significant in the multivariate analysis. Educational attainment has long been recognized as a protective factor by the World Bank and since 2004 by the Global Coalition On Women and AIDS (UNAIDS Initiative) which have advocated for the exemption of school fees and the encouragement of HIV prevention education in schools [69,70]. As a result, every Swazi child is entitled to free primary school education in public schools. This policy has obvious positive outcomes as 95.3% of girls of schooling age are now able to read and write [71]. However, our results suggested that keeping girls in school only until primary education may still be insufficient to reduce the risk of HIV infection and further suggesting the amendment of national policy to safeguard girls’ school enrollment until high school. Moreover, though enrolled in the education system, as much as 55% of participants reported to have dropped out due to lack of financial support (30%, S2 Table). As reviewed by Hardee and colleagues, girls face numerous barriers to stay in school such as lack of money to buy uniforms and textbooks. In addition, inadequate sanitary facilities also discourage girls to attend school especially during menstruation [19]. Such poor attendance may lead to low academic performance resulting in dropouts later on. Efforts should ensure not only to encourage higher educational attainment but also the uninterrupted school attendance among Swazi population, particular the girls, as such interventions have shown effectiveness in HIV risk reduction in the neighboring South Africa [28].

Regarding economic factors, ecological indices such as the Gross National Income has been shown to be inversely related to national HIV prevalence in SSA [72]. Similarly, at the individual level, a higher HIV prevalence is well documented in women with lower economic status [21,73,74]. In addition, it is evident that economic empowerment and cash transfer interventions targeting women have resulted in lower risky sexual behaviors [19,20,75]. Furthermore, a recent analysis in South Africa showed that cash or cash-in-kind reduced HIV risk among girls by mitigating pathways of poverty that increased their vulnerability [76]. While there is plenty of anecdotal evidence suggesting a link between poverty and HIV in Swaziland, empirical evidence from studies with methodological rigor are limited [10,77] prior to our study. Though Miller and colleagues identified models of transactional sex in Swaziland indicating possible mechanisms through which low income might lead to HIV risk, the research is not an epidemiological study [78]. In our study, we found a clear dose-dependent relationship between lower economic status and HIV infection with both current cash income and childhood household wealth index. While the latter index lost statistical significance in the multivariate analysis probably because of the relatively strong association it had with level of education (r = 0.39), childhood household wealth index may contribute to HIV vulnerability through poor educational attainment. In other words, while current low income may directly put women in socially vulnerable situation to HIV infection, childhood household wealth status may also affect HIV infection through limited education opportunities. However, further research should seek to identify these mechanisms to design appropriate interventions relevant to the Swazi context. We hope that this evidence will allow for better prioritization of HIV prevention interventions that focus on economic empowerment of women.

### Sexual behavior-related factors

Many of the sexual-related factors identified to increase the risk of HIV infection in this study have been well documented in previous studies in many countries including those in SSA. In our study, ≥2 lifetime number of sexual partners was the most prevalent (75%) and a powerful predictor of HIV infection (AOR > 3). It is important to note that half of the women who had MSP cited financial benefit as a reason; strongly suggesting that poverty perpetuates the practice of MSP in Swaziland. Ever cohabiting was also found to be a strong predictor of HIV infection (AOR > 2) and associated with the highest HIV prevalence (53%). In recent years, cohabiting is on the rise in Swaziland due to the inability of men to pay bridal payment (dowry) as a pre-requisite of marriage (a practice prominent in Swaziland) leading men to cohabit with multiple women for longer period of time, thus increasing unprotected coital frequency which results in an increased risk of HIV infection [9,79]. An alarming finding in the Swazi context, is the fact that 75% and 30% of women had first sex and last sex respectively without knowing their partner’s HIV status and had an elevated risk for HIV infection (AOR = 1.6 and AOR = 1.5). As a country with a highly generalized HIV epidemic, as high as 30–40% on average in both men and women [6], revitalization of campaigns to promote safe sex with a partner of unknown HIV status, as well as support programs to encourage couple testing and HIV status disclosure should be prioritized.

### HIV/AIDS-related knowledge

Finally, HIV/AIDS-related knowledge level was generally high: 80–90% of respondents correctly identified that a healthy looking person can be HIV positive, the risk of HIV infection can be reduced by avoiding MSP and using condoms. This suggests that young women in Swaziland are engaging in HIV risky behaviors not because of lack of knowledge. Due to the cross-sectional nature of our study, it could be argued that respondents may have recently gained HIV/AIDS-related knowledge during recent antenatal care visits and thus, their past risky sexual behaviors were primarily due to lower knowledge levels prior to antenatal checkups. Nonetheless, our data does not support this view since only 9.4% of respondents reported “lack of knowledge of HIV risks” as a reason for MSP. Furthermore, high HIV/AIDS-related knowledge has been previously reported in national surveys; e.g. 80–90% of women in the Swaziland Demographic Health Survey (2007) correctly identified ways to reduce HIV infection[40]. This is also consistently true among all age ranges, counter-arguing the concern that young people may not have had adequate information before their sexual debut, hence, thrusting them into risky behaviors. Data from the Multiple Indicator Cluster Survey (2010) is in concordance, demonstrating that the general public is well-equipped with adequate knowledge [41]. For these reasons, risky behaviors are unlikely due to lack of knowledge but most likely because of low income and low educational attainment as discussed above. As demonstrated by our findings, the gap between knowledge and practice is yet of great concern. The Extended National Multisectoral HIV and AIDS Framework has pointed this out by stating that “HIV and AIDS awareness and knowledge has not translated into the desired levels of behavior change due to inadequate personal risk perception that focus on translating knowledge into action” [10] noted in 2012. As the gap is still largely predominant in our findings, therefore the country urgently needs more innovative strategies and revitalization of existing ones because interventions centered on HIV/AIDS-related knowledge alone may not be sufficient to deter women from engaging in HIV risky sexual behavior.

### Strengths and limitations

This study was designed to maximize internal and external validity. First, the study was conducted in the region where HIV prevalence among pregnant women is highest. Second, simple two-stage cluster sampling was adopted to ensure the representativeness of pregnant women with a systematic effort to maximize response rate (92%). Third, appropriate statistical procedures were adopted to adjust for clustering effect on the variances of point estimates. Fourth, the study was conducted using self-administered questionnaire with internet-enabled tablet devices to minimize interviewer bias and socially desirable responses on the sensitive issues of HIV status, income and sexual behavior. In spite of these efforts, this study has some limitations. First, recall bias could have been introduced since our questionnaire asked retrospective factors such as first sex and childhood household belongings. Second, contamination of socially desirable answer is still possible to sensitive questions. Third, cause-effect relationship cannot be inferred due to its cross-sectional nature. Lastly, this study may not fully represent all women of reproductive age in Swaziland since women using contraceptives were not included therefore, the generalization of these findings should be done with caution.

## Conclusion

Family function did not appear to increase the risk for self-reported HIV status among pregnant women attending antenatal care in our study. However, given the scarcity of studies exploring the role of family function in the specific context of the Swazi HIV epidemic, we recommend further studies. Taken altogether, our study showed that risky sexual behavior was unlikely due to the lack of HIV/AIDS-related knowledge but due to structural factors such as education and economic situation. Therefore, besides programs that promote HIV knowledge and safer sexual practice, interventions that address structural factors by ensuring opportunities for higher education and by providing sustainable financial support to young women should be promoted.

## Supporting Information

S1 Table Descriptive and bivariate statistics for HIV related knowledge items. Descriptive and bivariate statistics for HIV/AIDS related knowledge items associated with self-reported HIV infection. Complex SE = Standard error of estimate under complex sampling analysis. DEFF = Design effect. P value calculated using the second-order Rao-Scott adjusted chi-square statistic
(DOCX)

S2 Table Descriptive frequency statistics for reason of dropping out of school. This table shows distribution of reasons for dropping out of school and self-reported HIV infection.*Where excluded because they were considered too young to reliably know the reason for dropping out of school since they did not complete primary school education
(DOCX)

S1 Dataset Dataset of this study, (SAV)

S1 Questionnaire siSwati and English version of the questionnaire. (DOCX)

## References

[1] World Health Organization. Women’s Health Fact Sheet [Internet]. World Health Organization Media Centre 2014 [cited 2016 Apr 8]. p. 1–9. Available from: http://www.who.int/mediacentre/factsheets/fs334/en/

[2] UNAIDS. Adolescent girls and young women and HIV/AIDS [Internet]. UNAIDS 2014 [cited 2016 Apr 1]. p. 1–14. Available from: http://www.unaids.org/sites/default/files/media_asset/02_Adolescentgirlsandyoungwomen.pdf

[3] National AIDS Control Council. Kenya HIV Estimates 2014 [Internet]. Ministry of Health Kenya 2014 [cited 2016 Apr 8]. p. 1–28. Available from: http://reliefweb.int/sites/reliefweb.int/files/resources/HIVestimatesreportKenya2014_print.pdf

[4] Republic of Namibia Ministry of Health and Social Services. The Namibia AIDS response progress report 2015 [Internet]. 2015 [cited 2016 Apr 8]. Available from: http://www.unaids.org/sites/default/files/country/documents/NAM_narrative_report_2015.pdf

[5] Statistics South Africa. South Africa mid-year population estimates 2015. [Internet]. 2015 [cited 2016 Apr 8]. Available from: https://www.statssa.gov.za/publications/P0302/P03022015.pdf

[6] Bicego GT, Nkambule R, Peterson I, Reed J, Donnell D, Ginindza H, et al Recent Patterns in Population-Based HIV Prevalence in Swaziland. PLoS One. 2013;8(10):e77101 10.1371/journal.pone.0077101 24143205 PMC3797108

[7] Mngadi S, Fraser N, Mkhatshwa H, Lapidos T, Khumalo S, Nhlabatsi N, et al Swaziland HIV Prevention Response and Modes of Transmission Analysis [Internet]. National Emergency Council on HIV and AIDS (NERCHA) 2009 [cited 2012 Jan 8]. Available from: http://www.k4health.org/sites/default/files/Swaziland_MoT_Country_Synthesis_Report_22Mar09.pdf

[8] UNAIDS. How Africa turned AIDS around [Internet]. UNAIDS Special report. 2013 [cited 2016 Apr 20]. p. 1–52. Available from: http://www.unaids.org/sites/default/files/media_asset/20130521_Update_Africa_1.pdf

[9] Ramjee G, Daniels B. Women and HIV in Sub-Saharan Africa. AIDS Res Ther. 2013;10(1):30 10.1186/1742-6405-10-30 24330537 PMC3874682

[10] National Emergence Response Council on HIV and AIDS (NERCHA). The Extended National Multisectoral HIV and AIDS Framework (eNSF) 2014–2018 [Internet]. Mbabane: National Emergence Response Council on HIV and AIDS (NERCHA); 2014 [cited 2016 Mar 20]. Available from: http://hivhealthclearinghouse.unesco.org/sites/default/files/resources/swaziland_ensf_hiv-aids_2014_2018.pdf

[11] Pequegnat W, Bell CC. Family and HIV/AIDS: Cultural and Contextual Issues in Prevention and Treatment. Pequegnat W, Bell CC, editors. Springer. London: Springer New York Dordrecht Heidelberg London; 2012. 1–364 p.

[12] Voisin DR. Family ecology and HIV Sexual Risk Behaviors Among African American and Perto Rican Adolescent Males. Am J Orthopsychiatry. 2002;72(2):294–302. 15792069 10.1037/0002-9432.72.2.294

[13] Bell CC, Bhana A, Petersen I, Mckay MM. Building Protective Factors to Offset Sexually Risky Behaviors among Black Youths: A Randomized Control Trial. J Natl Med Assoc. 2008;100(8):936–44. 18717144 10.1016/s0027-9684(15)31408-5 PMC2536567

[14] Wamoyi J, Fenwick A, Urassa M, Zaba B, Stones W. Parent-child communication about sexual and reproductive health in rural Tanzania: Implications for young people’s sexual health interventions. Reprod Health. 2010;7:6 10.1186/1742-4755-7-6 20462413 PMC2875205

[15] Wamoyi J, Wight D, Remes P. The structural influence of family and parenting on young people’s sexual and reproductive health in rural northern Tanzania. Cult Health Sex. 2015;17(6):718–32. 10.1080/13691058.2014.992044 25597368 PMC4419469

[16] Wamoyi J, Wight D. “Mum never loved me.” How structural factors influence adolescent sexual and reproductive health through parent-child connectedness: a qualitative study in rural Tanzania. African J AIDS Res. 2014;13(2):169–78. 10.2989/16085906.2014.945387 PMC4151808 25174634

[17] International Labour Office. BRIEF OCTOBER 2005 HIV / AIDS and poverty: the critical connection [Internet]. International Labour Office 2005 [cited 2016 Mar 17]. Available from: http://www.ilo.org/wcmsp5/groups/public/—ed_protect/—protrav/—ilo_aids/documents/publication/wcms_120468.pdf

[18] Parkhurst JO. Understanding the Correlations Between Wealth, Poverty and Human Immunodeficiency Virus Infection in African Countries. Bull World Health Organ. 2010;88(7):519–26. 10.2471/BLT.09.070185 20616971 PMC2897986

[19] Hardee K, Gay J, Croce-Galis M, Peltz A. Strengthening the enabling environment for women and girls: What is the evidence in social and structural approaches in the HIV response? J Int AIDS Soc. 2014;17:1–12. 10.7448/IAS.17.1.18619 PMC3887370 24405664

[20] Piot P, Abdool Karim SS, Hecht R, Legido-Quigley H, Buse K, Stover J, et al The Lancet Commissions A UNAIDS–Lancet Commission on Defeating AIDS—Advancing Global Health Defeating AIDS—advancing global health. Lancet. 2015;386(9989):171–218. 10.1016/S0140-6736(15)60658-4 26117719

[21] Mbirimtengerenji ND. Is HIV/AIDS epidemic outcome of poverty in sub-saharan Africa? Croat Med J. 2007;48(5):605–17. 17948947 PMC2205968

[22] Joint United Nations Programme on HIV/AIDS (UNAIDS). Women Out Loud: How Women Living With HIV Will Help The World End AIDS. [Internet]. UNAIDS 2012 [cited 2016 May 21]. p. 1–100. Available from: http://www.unaids.org/en/resources/presscentre/featurestories/2012/december/20121211womenoutloud

[23] Buvé A, Bishikwabo-Nsarhaza K, Mutangadura G. The spread and effect of HIV-1 infection in sub-Saharan Africa. Lancet. 2002;359(9322):2011–7. 10.1016/S0140-6736(02)08823-2 12076570

[24] Pettifor AE, van der Straten A, Dunbar MS, Shiboski SC, Padian NS. Early age of first sex: a risk factor for HIV infection among women in Zimbabwe. AIDS. 2004;18(10):1435–42. 15199320 10.1097/01.aids.0000131338.61042.b8

[25] Ma Q, Ono-Kihara M, Cong L, Xu G, Pan X, Zamani S, et al Early initiation of sexual activity: a risk factor for sexually transmitted diseases, HIV infection, and unwanted pregnancy among university students in China. BMC Public Health. 2009 1;4(9):111. 10.1186/1471-2458-9-111 PMC2674603 19383171

[26] Pettifor AE, O’Brien K, Macphail C, Miller WC, Rees H V. Early coital debut and associated HIV risk factors among young women and men in South Africa. Int Perspect Sex Reprod Health. 2009;35(2):82–90. 10.1363/ifpp.35.082.09 19620092

[27] Heywood W, Patrick K, Smith AMA, Pitts MK. Associations Between Early First Sexual Intercourse and Later Sexual and Reproductive Outcomes: A Systematic Review of Population-Based Data. Arch Sex Behav. 2015;44(3):531–69. 10.1007/s10508-014-0374-3 25425161

[28] Hargreaves JR, Morison L a, Kim JC, Bonell CP, Porter JDH, Watts C, et al The association between school attendance, HIV infection and sexual behaviour among young people in rural South Africa. J Epidemiol Community Health. 2008;62(2):113–9. 10.1136/jech.2006.053827 18192598

[29] Fox J, Fidler S. Sexual transmission of HIV-1. Antiviral Res. 2010 1;85(1):276–85. 10.1016/j.antiviral.2009.10.012 19874852

[30] Ahmed S, Lutalo T, Wawer MJ, Serwadda DM, Sewankambo NK, Nalugoda FK, et al HIV incidence and sexually transmitted disease prevalence associated with condom use: a population study in Rakai, Uganda. Aids. 2001;15(16):2171–9. 11684937 10.1097/00002030-200111090-00013

[31] Morris M, Kretzschmar M. Concurrent partnerships and the spread of HIV. AIDS. 1997 4;11(5):641–8. 9108946 10.1097/00002030-199705000-00012

[32] Sawers L. Review article Measuring and modelling concurrency. J Int AIDS Soc. 2013;16:1–20. 23406964 10.7448/IAS.16.1.17431 PMC3572217

[33] Mercer CH, Aicken CRH, Tanton C, Estcourt CS, Brook MG, Keane F, et al Serial monogamy and biologic concurrency: Measurement of the gaps between sexual partners to inform targeted strategies. Am J Epidemiol. 2013;178(2):249–59. 10.1093/aje/kws467 23801013

[34] Chersich MF, Rees H V. Vulnerability of women in southern Africa to infection with HIV: biological determinants and priority health sector interventions. AIDS. 2008;22 Suppl 4:S27–40. 10.1097/01.aids.0000341775.94123.75 19033753

[35] Kalichman SC, Simbayi LC, Kaufman M, Cain D, Jooste S. Alcohol use and sexual risks for HIV/AIDS in sub-Saharan Africa: systematic review of empirical findings. Prev Sci. 2007 6;8(2):141–51. 10.1007/s11121-006-0061-2 17265194

[36] Lama TP, Kumoji E ‘Kuor, Ketlogetswe D, Anderson M, Brahmbhatt H. Alcohol Consumption and Risky Sexual Behavior Among Persons Attending Alcohol Consumption Venues in Gaborone, Botswana. Prev Sci. 2016;17(2):227–36. 10.1007/s11121-015-0607-2 26450847

[37] Risher K, Adams D, Sithole B, Ketende S, Kennedy C, Mnisi Z, et al Sexual stigma and discrimination as barriers to seeking appropriate healthcare among men who have sex with men in Swaziland. J Int AIDS Soc. 2013 1;16(3 Suppl 2):18715. 24242263 10.7448/IAS.16.3.18715 PMC3833105

[38] Fielding-Miller R, Mnisi Z, Adams D, Baral S, Kennedy C. “There is hunger in my community”: a qualitative study of food security as a cyclical force in sex work in Swaziland. BMC Public Health. BMC Public Health; 2014 1;14(1):79. 24460989 10.1186/1471-2458-14-79 PMC3905928

[39] Sacolo HN, Chung M-H, Chu H, Liao Y-M, Chen C-H, Ou K-L, et al High risk sexual behaviors for HIV among the in-school youth in Swaziland: a structural equation modeling approach. PLoS One. 2013 1;8(7):e67289 10.1371/journal.pone.0067289 23861756 PMC3701534

[40] Central Statistical Office (CSO) [Swaziland] and Macro Internationa Inc. Swaziland Demographic and Health Survey 2006–07 [Internet]. Demographic and Health Surveys. Mbabane: Central Statistical Office (CSO) [Swaziland] and Macro Internationa Inc; 2007 [cited 2015 Oct 20]. Available from: http://dhsprogram.com/pubs/pdf/fr202/fr202.pdf

[41] Central Statistical Office and UNICEF. Swaziland Multiple Indicator Cluster Survey 2011 [Internet]. Central Statistical Office and UNICEF 2011 [cited 2016 Jan 20]. Available from: http://reliefweb.int/sites/reliefweb.int/files/resources/MICS4_Swaziland_FinalReport_2010_Eng.pdf

[42] Nkhambule M, Mbingo S, Malinga M. HIV Prevention: Multiple and Concurrent Sexual Partnerships among Youth and Adults in Swaziland. 2008.

[43] National Emergency Response Council on HIV/AIDS. Swaziland Hearsay Ethnography Study Final draft report [Internet]. National report. 2011 [cited 2016 Jan 20]. Available from: https://www.k4health.org/sites/default/files/HearsayEthnography.pdf

[44] Ruark A, Dlamini L, Mazibuko N, Green C. Love, Lust, and the Emotional Context of Concurrent Sexual Partnerships among Young Swazi Adults. African J AIDS Res. 2014;13(2):133–43. 10.2989/16085906.2014.927781 PMC4201849 25174630

[45] Ministry of Health Swaziland. 12th National HIV Serosurvellance among Women Attending Antenatal Care Services in Swaziland. 2011.

[46] Schroder KEE, Carey MP, Vanable PA, Ph D, Carey MP, Vanable PA, et al Methodological challenges in research on sexual risk behavior: II. Accuracy of self-reports. Ann Behav Med. 2003;26(2):104–23. 14534028 10.1207/s15324796abm2602_03 PMC2441938

[47] Techasrivichien T, Darawuttimaprakorn N, Punpuing S, Musumari PM, Lukhele BW, El-saaidi C, et al Changes in Sexual Behavior and Attitudes Across Generations and Gender Among a Population-Based Probability Sample From an Urbanizing Province in Thailand. Arch Sex Behav. 2014;45(2):367–82. 10.1007/s10508-014-0429-5 25403321 PMC4706588

[48] Central Intelligence Agency. The world factbook: Country Comparison to the World [Internet]. Central Intelligence Agency 2011 [cited 2016 Apr 25]. Available from: https://www.cia.gov/library/publications/the-world-factbook/fields/2256.html

[49] Central Statistical Office. Swaziland Multiple Indicator Cluster Survey: Key Findings [Internet]. Central Statistical Office 2015 [cited 2016 May 2]. Available from: https://mics-surveys-prod.s3.amazonaws.com/MICS5/EasternandSouthernAfrica/Swaziland/2014/Keyfindings/Swaziland2014MICSKFR_English.pdf

[50] Ministry of Health. Service Availability Mapping Report 2013. Swaziland Government. 2013.

[51] Michael Kohn S.., Jarrett S Michael SJ. Sample size calculators: Sample Size Calculators for Designing Clinical Research [Internet]. University of California, San Francisco 2015 [cited 2014 Feb 2]. p. about 2 screens. Available from: http://www.sample-size.net/sample-size-proportions/

[52] Hulley Stephen B., Cummings Steven R., Browner Warren S., Deborah G. Grady TBN. Designing Clinical Research. 3rd ed. Philadelphia: Lippincott Williams & Wilkins; 2007.

[53] Huebner AJ, Howell LW. Examining the relationship between adolescent sexual risk-taking and perceptions of monitoring, communication, and parenting styles. J Adolesc Health. 2003;33(2):71–8. 12890597 10.1016/s1054-139x(03)00141-1

[54] Family Health International. Behavioral Surveillance Surveys: Guidelines for repeated Behavioral surveys in population at risk of HIV [Internet]. Family Health International 2000 [cited 2016 Jan 14]. Available from: http://www.who.int/hiv/strategic/en/bss_fhi2000.pdf

[55] Creswell JW PC. Advanced Mixed Methods Research Designs. Tashakki A TC, editor. Thousand Oaks Sage Publications; 2003. 209–240 p.

[56] Olson DH, Gorall DM, Tiesesl JW. Family Adaptability and Cohesion Evaluation Scales (FACES IV). Life innovations, Inc. 2004.

[57] Paul S. Levy SL. Sampling of Populations: Methods and Applications, 4th Edition 4th ed. New Jersey: John Wiley & Sons, Inc.,Hoboken; 2008.

[58] Sullivan PS, Karon JM, Malitz FE, Broyles S, Mokotoff ED, Buskin SE, et al A two-stage sampling method for clinical surveillance of individuals in care for HIV infection in the United States. Public Health Rep. 2005;120(3):230–9. 16134562 10.1177/003335490512000304 PMC1497713

[59] Kish L. Survey Sampling. Wikey-Interscience Publication; 1995.

[60] Vyas S, Kumaranayake L. Constructing socio-economic status indices: How to use principal components analysis. Health Policy Plan. 2006;21(6):459–68. 10.1093/heapol/czl029 17030551

[61] Fry K, Firestone R, Chakraborty NM. Measuring Equity with Nationally Representative Wealth Quintiles. Washington DC; 2014.

[62] DiClemente RJ, Wingood GM, Crosby R, Sionean C, Cobb BK, Harrington K, et al Parental Monitoring: Association With Adolescents’ Risk Behaviors. Pediatrics. 2001 6 1;107(6):1363–8. 11389258 10.1542/peds.107.6.1363

[63] Thoma BC, Huebner DM. Parental Monitoring, Parent–Adolescent Communication About Sex, and Sexual Risk Among Young Men Who Have Sex with Men. AIDS Behav. 2014 8 19;18(8):1604–14. 10.1007/s10461-014-0717-z 24549462 PMC4108561

[64] Markham CM, Lormand D, Gloppen KM, Peskin MF, Flores B, Low B, et al Connectedness as a Predictor of Sexual and Reproductive Health Outcomes for Youth. J Adolesc Heal. Elsevier Inc; 2010;46(3 SUPPL):S23–41. 10.1016/j.jadohealth.2009.11.214 20172458

[65] Armistead L, Cook S, Skinner D, Toefy Y, Anthony ER, Zimmerman L, et al Preliminary results from a family-based HIV prevention intervention for South African youth. Heal Psychol. 2014;33(7):668–76. 10.1037/hea0000067 24977310

[66] Crosby RA, DiClemente RJ, Wingood GM, Lang DL, Harrington K. Infrequent parental monitoring predicts sexually transmitted infections among low-income African American female adolescents. Arch Pediatr Adolesc Med. 2003;157(2):169–73. 12580687 10.1001/archpedi.157.2.169

[67] UNDP Swaziland. Swaziland Human Development Report: HIV and AIDS and Culture [Internet]. United Nations Development Programme 2007 [cited 2016 Apr 9]. Available from: http://hdr.undp.org/sites/default/files/swaziland_nhdr_2008.pdf

[68] Hammond RJ, Cheney P. SOCIOLOGY OF THE FAMILY. 2010.

[69] The Global Coalition on Women and AIDS. Educate Girls Fight AIDS [Internet]. UNAIDS 2005 [cited 2016 Mar 15]. p. 4 Available from: http://data.unaids.org/GCWA/gcwa_fs_girlseducation_sep05_en.pdf

[70] The World Bank. HIV/AIDS and Education [Internet]. The World Bank; [cited 2016 May 14]. Available from: http://go.worldbank.org/AWN1CO49D0

[71] UNICEF. Statistics | Swaziland | UNICEF [Internet]. UNICEF. 2013 [cited 2016 Jan 25]. Available from: http://www.unicef.org/infobycountry/swaziland_statistics.html

[72] Fox AM. “The Social Determinants of HIV Serostatus in Sub-saharan Africa: An Inverse Relationship Between Poverty and HIV?” Public Health Rep. 2010;125:16–24. 10.1177/00333549101250S405 PMC2882971 20629252

[73] Masanjala W. The poverty-HIV/AIDS nexus in Africa: A livelihood approach. Soc Sci Med. 2007;64(5):1032–41. 10.1016/j.socscimed.2006.10.009 17126972

[74] Magadi M a. The disproportionate high risk of HIV infection among the urban poor in sub-Saharan Africa. AIDS Behav. 2013;17(5):1645–54. 10.1007/s10461-012-0217-y 22660933 PMC3663197

[75] Handa S, Halpern CT, Pettifor A, Thirumurthy H. The Government of Kenya’s cash transfer program reduces the risk of sexual debut among young people age 15–25. PLoS One. 2014;9(1). 10.1371/journal.pone.0085473 PMC3893206 24454875

[76] Cluver LD, Orkin FM, Meinck F, Boyes ME, Sherr L. Structural drivers and social protection: mechanisms of HIV risk and HIV prevention for South African adolescents. J Int AIDS Soc. 2016;19(1):20646 10.7448/IAS.19.1.20646 27086838 PMC4834365

[77] Whiteside A, Hickey A, Ngcobo N, Tomlinson J. What is driving the HIV / AIDS epidemic in Swaziland, and what more can we do about it? Final report prepared. 2003;(April).

[78] Fielding-Miller R, Dunkle KL, Cooper HLF, Windle M, Hadley C. Cultural Consensus Modeling to Measure Tansactional Sex in Swaziland: Scale Building and Validation. Soc Sci Med. Elsevier Ltd; 2016 1;148:25–33. 10.1016/j.socscimed.2015.11.024 26647365 PMC4698209

[79] Dunkle KL, Stephenson R, Karita E, Chomba E, Kayitenkore K, Vwalika C, et al New heterosexually transmitted HIV infections in married or cohabiting couples in urban Zambia and Rwanda: an analysis of survey and clinical data. Lancet. 2008;371(9631):2183–91. 10.1016/S0140-6736(08)60953-8 18586173

