# Supplementary material for: Structural and Behavioral Correlates of HIV Infection among Pregnant Women in a Country with a Highly Generalized HIV Epidemic: A Cross-Sectional Study with a Probability Sample of Antenatal Care Facilities in Swaziland
Source: PLoS One. 2016 Dec 12;11(12):e0168140. doi: 10.1371/journal.pone.0168140 (PMC5152904; doi:10.1371/journal.pone.0168140)
Supplement: S1 Table — Descriptive and bivariate statistics for HIV/AIDS related knowledge items associated with self-reported HIV infection. Complex SE = Standard error of estimate under complex sampling analysis. DEFF = Design effect. P value calculated using the second-order Rao-Scott adjusted chi-square statistic (DOCX) [file pone.0168140.s001.docx]

S1 Table. Descriptive and bivariate statistics for HIV/AIDS related knowledge items associated with self- reported HIV infection

|  |  | Total  N=827 | % of total | HIV Positive | % HIV Positive | Complex SE | DEFF | P value | |
| --- | --- | --- | --- | --- | --- | --- | --- | --- | --- |
| **HIV Knowledge** |  |  |  |  |  |  |  |  | |
| Can people reduce their chances of getting HIV  by having one uninfected sexual partner? |  |  |  |  |  |  |  |  | |
|  | Yes | 692 | 83.7 | 240 | 34.9 | 2.5 | 2.41 |  | |
|  | No | 135 | 16.3 | 295 | 40.7 | 4.9 | 1.69 | 0.35 | |
| Can people get HIV from mosquito bites? |  |  |  |  |  |  |  |  | |
|  | Yes | 137 | 16.6 | 50 | 38.0 | 4.2 | 1.28 | 0.69 | |
|  | No | 690 | 83.4 | 245 | 35.5 | 2.8 | 2.87 |  | |
| Can people reduce their chances of getting HIV  by using a condom every time they have sex? |  |  |  |  |  |  |  |  | |
|  | Yes | 736 | 89.0 | 273 | 37.1 | 2.3 | 2.08 |  | |
|  | No | 91 | 11.0 | 24 | 26.4 | 3.3 | 0.618 | 0.03 | |
| Can people reduce their chances of getting HIV  by abstaining from sex? |  |  |  |  |  |  |  | |  |
|  | Yes | 714 | 86.3 | 258 | 36.1 | 2.4 | 2.26 |  | |
|  | No | 113 | 13.7 | 39 | 34.5 | 3.4 | 0.70 | 0.71 | |
|  |  |  |  |  |  |  |  |  | |
| Can people get HIV through witchcraft? |  |  |  |  |  |  |  |  | |
|  | Yes | 63 | 7.6 | 26 | 41.3 | 4.5 | 0.67 | 0.27 | |
|  | No | 764 | 92.4 | 271 | 35.5 | 2.2 | 2.03 |  | |
|  |  |  |  |  |  |  |  |  | |
| Can people get HIV from open wounds or sores  of an infected person? |  |  |  |  |  |  |  |  | |
|  | Yes | 760 | 91.9 | 279 | 36.7 | 2.2 | 1.90 |  | |
|  | No | 67 | 8.1 | 18 | 26.9 | 3.5 | 0.51 | 0.01 | |
| Is it possible for a healthy looking person to have HIV? |  |  |  |  |  |  |  |  | |
|  | Yes | 754 | 91.2 | 278 | 36.9 | 2.1 | 1.69 |  | |
|  | No | 73 | 8.8 | 19 | 26.0 | 4.7 | 1.02 | 0.38 | |
| Do you know a place where people can go to get tested for HIV? |  |  |  |  |  |  |  |  | |
|  | Yes | 794 | 96.0 | 289 | 36.4 | 2.4 | 2.39 |  | |
|  | No | 33 | 4.0 | 8 | 24.2 | 6.3 | 0.89 | 0.20 | |

Complex SE= Standard error of estimate under complex sampling analysis

DEFF= Design effect

P value calculated using the second-order Rao-Scott adjusted chi-square statistic
