# Supplementary material for: Structural and Behavioral Correlates of HIV Infection among Pregnant Women in a Country with a Highly Generalized HIV Epidemic: A Cross-Sectional Study with a Probability Sample of Antenatal Care Facilities in Swaziland
Source: PLoS One. 2016 Dec 12;11(12):e0168140. doi: 10.1371/journal.pone.0168140 (PMC5152904; doi:10.1371/journal.pone.0168140)
Supplement: S2 Table — This table shows distribution of reasons for dropping out of school and self-reported HIV infection.*Where excluded because they were considered too young to reliably know the reason for dropping out of school since they did not complete primary school education (DOCX) [file pone.0168140.s002.docx]

|  |  | Total  N=827 | % of total | HIV Positive | % HIV Positive |
| --- | --- | --- | --- | --- | --- |
| Reason of dropping out of school | Fell pregnant | 129 | 15.6 | 49 | (38.0) |
|  | Found a boyfriend | 6 | 0.7 | 2 | (33.3) |
|  | Got married | 13 | 1.6 | 3 | (23.1) |
|  | Did not have financial support | 245 | 29.6 | 120 | (49.0) |
|  | Did not do well at school | 20 | 2.4 | 6 | (30.0) |
|  | Other | 13 | 1.6 | 6 | (46.2) |
|  | Excluded* | 26 | 3.1 | 13 | (50.0) |

**S2 Table. Descriptive frequency statistics for reason of dropping out of school.**

*Where excluded because they were considered too young to reliably know the reason for dropping out of school since they did not complete primary school education.
