# Supplementary material for: Structural and Behavioral Correlates of HIV Infection among Pregnant Women in a Country with a Highly Generalized HIV Epidemic: A Cross-Sectional Study with a Probability Sample of Antenatal Care Facilities in Swaziland
Source: PLoS One. 2016 Dec 12;11(12):e0168140. doi: 10.1371/journal.pone.0168140 (PMC5152904; doi:10.1371/journal.pone.0168140)
Supplement: S1 Questionnaire — (DOCX) [file pone.0168140.s004.docx]

**IMIBUTO NGELULWIMI LWESISWATI**

| **Ligama lesibhedlela:** | | | | | | | |  |  |
| --- | --- | --- | --- | --- | --- | --- | --- | --- | --- |
| **#** | | **Umbuto** | **Imphendvulo** | | | | |  |  |
| **Umniningwane yemuntfu** | | | | | | | |  |  |
| Q1 | | Uneminyaka lemingakhi? |  | | | | |  |  |
| Q2 | | Ushadile noma wendzile yini? | 1. Tange ngendze phambilini 2. Ngendzile 3. Hlala namasihlalisane 4. Sicabene asihlali ndzawonye 5. Sehlukene 6. Ngumfelokati | | | | |  |  |
| Q2b | | Ngaphandle kwakho, indvodza yakho inabo labanye bafati?  Buta labatsatsene bodvwa | 1. Yebo  2. Cha | | | | |  |  |
| Q3 | | Gcine kabana esikolweni?  Shano kutsi ugcine kabana, sibonelo usho kutsi ngagcine ebangeni lesine. | 1. Zange ngize ngiye esikolweni 2. Libanga lemfundvo lephasi 3. Libanga lemfundvo lelisemkhatsini 4. Libanga lemfundvo lephakeme 5. Ngagcina ekolishi | | | | |  |  |
| Q4 | | While you were a school going child have you ever stayed at a boarding school (hostel)?  Wake wahlala esikolweni usafundza | 1. Yebo 2. Chake | | | | |  |  |
| Q5 | | Wake wayekela esikolweni? | 1. Yebo 2. Chake…………………………………..Yani kumbuto 7 | | | | |  |  |
| Q6 | | Uma wake wayekela esikolweni shano kutsi wayekela kabana?  Uma kunetizatfu letinengi ketsa leso lesimatima kunato tonkhe | 1. Ngakhulelwa 2. Ngatfola lisoka 3. Ngendza 4. Bekute longangifundzisa 5. Angentanga kahle esikolweni 6. Letinye tizatfu tichaze…………………………………………………………………….. | | | | |  |  |
| Q7 | | What is your current employment status?  Yini umsebenti wakho manje? | 1. Angisebenti 2. Angikacashwa 3. Ngisafundza 4. Ngiyatisebenta | | | | |  |  |
| Q8 | | Lomdeni lokhulele kuwo unato yini letitntfo leti tasendlini?  (uma uke washintja imindeni/emakhaya shano lendzawo lohleli kuyo kakhulu ibe sibonelo)  (Mhlawumbe umndeni wakho washintja simo sawo usakhula, yenta leso simo lesitsatse sikhatsi lesidze sibe sibonelo) | Yebo Chake   \| 1. Gesi \| ① \| ⓪ \|  \| \| --- \| --- \| --- \| --- \| \| 1. Iwayilesi \| ① \| ⓪ \|  \| \| 1. Mabonakudze \| ① \| ⓪ \|  \| \| 1. Mahlale khikhini \| ① \| ⓪ \|  \| \| 1. Lucingo lasendlini \| ① \| ⓪ \|  \| \| 1. Sicandzisa kudla \| ① \| ⓪ \|  \| \| 1. Sitofu \| ① \| ⓪ \|  \| \| 1. Liwashi \| ① \| ⓪ \|  \| | | | | |  |  |
| Q9 | | Sitofu besisentjetiswa ekhaya kini besiluhloboni?  (Mhlawumbe ushintje tindzawo tekuhlala shano lendzawo lehleti kuyo sikhatsi lesidze sibonelo)  (Mhlawumbe umndeni wakho washintja simo sawo usakhula, yenta leso simo lesitsatse sikhatsi lesidze sibe sibonelo) | Yebo Chake   \| 1. Sitofu sagesi \| ① \| ⓪ \|  \| \| --- \| --- \| --- \| --- \| \| 1. Sitofu semalahle \| ① \| ⓪ \|  \| \| 1. Sitofu setinkhuni \| ① \| ⓪ \|  \| \| 1. Sitofu sahendigesi \| ① \| ⓪ \|  \| \| 1. Sitofu sapharafini \| ① \| ⓪ \|  \| \| 1. Sitofu semalahle \| ① \| ⓪ \|  \| | | | | |  |  |
| Q10 | | Kuke kwaba khona yini emndenini wakini loba nelibhayisikili?  (Mhlawumbe ushintje tindzawo tekuhlala shano lendzawo lehleti kuyo sikhatsi lesidze sibonelo)  (Mhlawumbe umndeni wakho washintja simo sawo usakhula, yenta leso simo lesitsatse sikhatsi lesidze sibe sibonelo | Yebo Chake   \| 1. Libhayisikili \| ① \| ⓪ \|  \| \| --- \| --- \| --- \| --- \| \| 1. Sidudu noma sikuta \| ① \| ⓪ \|  \| \| 1. Umgugugu \| ① \| ⓪ \|  \| \| 1. Imoto noma iloli \| ① \| ⓪ \|  \| \| 1. Sigulumba \| ① \| ⓪ \|  \| | | | | |  |  |
|  |  |  |  | | | | |  |  |
| Q11 | | Imali lengena kuwe njalo ngenyanga kuto tonkhe tinhlangotsi ngumalini?  Uma ungasebenti ungaholi khetsa (ngephansi kwa E249) | 1. Ngephansi kwa E249 2. Ngetulu kwa E249 kepha ngephansi kwa E1749 3. Ngetulu kwa E1749 kepha ngephansi kwa E3000 4. Ngetulu kwa 3000 | | | | |  |  |
| Q12 | | Usengaphansi kwemnyaka welishumi nesiphohlongo wawuya kangaphi esontfweni? | 1. Onkhemalanga 2. Kanye ngeliviki 3. Lokungenani kanye ngenyanga 4. Lokungenani kanye ngemnyaka 5. Ngaphansi kwakanye ngemnyaka 6. Angizange | | | | |  |  |
| **Tinkhomba temindeni**     \| ① \| ② \| ③ \| ④ \| ⑤ \| \| --- \| --- \| --- \| --- \| --- \| \| **Angivumi kakhulu** \| **Angivumi** \| **Sengakacabangi** \| **Ngiyavuma nje** \| **Ngivuma mbamba** \| | | | | | | | |  |  |
| Lesigaba lesi simayelana nemlandvo wemndeni wakini kutsi beniphila kanjani lomunye lomunye usengephansi kweminyaka lelishumi nesiphohlongo. Cela ukhombise lizinga lesivumelwano noma kungavumelani kwakho nalamaphuzu langephansi ngekukhetsa linye lalamaphuzu langahle abe ngiwo. | | | | | | | |  |  |
| Q13 | | Khani ngisengephansi kweminyaka lelishumi nesiphohlongo umndeni wami wawuvana nebantfu bangephandle kakhulu kunatsi lamndenini. | |  | | | |  |  |
| Q14 | | Khani ngisengephansi kwemnyaka lelishumi nesiphohlongo besicitsa sikhatsi ndzawonye nemndeni wami. | |  | | | |  |  |
| Q15 | | Khani ngisengephansi kweminyaka lelishumi nesiphohlongo besingavani kahle nemndeni wami. | |  | | | |  |  |
| Q16 | | Khani ngingephansi kwemnyaka lelishumi nesiphohlongo umndeni wami besivana kakhulu. | |  | | | |  |  |
| Q17 | | Khani ngingephansi kweminyaka lelishumi nesiphohlongo umndeni wami bobalekelana bangasekelani etintfweni | |  | | | |  |  |
| Q18 | | Khani ngingephansi kwemnyaka lelishumi nesiphohlongo umndeni wami bosekelana ngetikhatsi tebundzima | |  | | | |  |  |
| Q19 | | Khani ngengephansi kwemnyaka lelishumi nesiphohlongo kwakungatfolakali kutsi ngubani lomele kwenta emisebenti yelikhaya emndenini wami | |  | | | |  |  |
| Q20 | | While I was less than 18 years old my family rarely did things together  Khani ngingephansi kweminyaka lelishumi nesiphohlongo umndeni wami wawungahlanganyeli ndzawonye nabenta tintfo | |  | | | |  |  |
| Q21 | | Munye kubatali bami unesingani | | 1. Yebo 2. Chake 3. Angati | | | |  |  |
| Q22 | | Babe wami unsitsembu? | | 1. Yebo 2. Chake 3. Angati | | | |  |  |
| **Kunakekela kwebatali**     \| ① \| ② \| ③ \| ④ \| ⑤ \| \| --- \| --- \| --- \| --- \| --- \| \| **Ngiyala mbamba** \| **Ngiyala nje** \| **Sengakacabangi** \| **Ngiyavuma nje** \| **Ngivuma mbamba** \| | | | | | | | |  |  |
| Lesigaba lesilandzelako simayelana nekunakekela kwebatali nawungephansi kweminyaka lelishumi nesiphohlongo budzala. Uma ungazange uphile imphilo yakho nebatali bakho usesemncane, yenta sibonelo ngemndeni lowakhulela kuwo. Vumela nalamaphuzu ngekukhetsa linye liphuze lelingaba ngilo. | | | | | | | |  |  |
| Q23 | | Nangingaphansi kwemnyaka lelishumi nesiphohlongo kwakinetincabekelwane letimatima uma wephule umtsetfo emndenini wami | |  | | |  |  |  |
| Q24 | | Khani ngingephansi kwemnyaka lelishumi nesiphohlongo nangivakashile ebusuku batali bami bebasuke bati kutsi ngikuphi | |  | | |  |  |  |
| Q25 | | Khani ngingephansi kweminyaka lelishumi nesiphohlongo ngangibhekeke kubatisa batali bami kutsi ngitobe nginabani.  Ikakhulu nangihambe nebangani | |  | | |  |  |  |
| Q26 | | Khani ngingephansi kweminyaka lelishumi nesiphohlongo bekunemtsetfo waso sonkhe sime emndenini wami | |  | | |  |  |  |
| Q27 | | Khani ngingaphansi kweminyaka lelishumi nesiphohlongo Batali bami nabenta sivumelwano bekulikhuni kusishintja | |  | | |  |  |  |
| Q28 | | Khani ngingephansi kwemnyaka lelishumi nesiphohlongo Batali bami bebati nangibuya emvakwelihora esontfweni/esikholweni noma ngukuphi lokunye | |  | | |  |  |  |
| **Kucoca kwebatali nebantfwana**   \| ① \| ② \| ③ \| ④ \| ⑤ \| \| --- \| --- \| --- \| --- \| --- \| \| **Angivumi kakhulu** \| **Angivumi nje** \| **Angikakhetsi** \| **Ngiyavuma nje** \| **Ngivuma kakhulu** \| | | | | | | | | |  |
| Lesicephu lesilandzelako simayelana nekukhulumisana kwebatali nebantfwana labangephansi kwelishumi nesiphohlongo. Uma ungazange uphile sikhatsi lesidze nebatali bakho mbamba ebuntfwaneni, yenta sibonelo ngesihlobo sakho lesidvuna simele babe wakho noma lesisikati lesimele make wakho. Shano lizinga lesivumelwano noma kungavumelani nalomubono longephansi ngekukhetsa sinye imphendvulo kuleti letisihlanu letilandzelako ngalinye liphuzu. | | | | | | | | |  |
| Q29 | Khani ngingephansi kweminyaka lelishumi nesiphohlongo bengeneliseka ngendlela bengikhulumisana ngayo nebatali bami esikhatsini lesinengi. | | | |  |  | | | |
| Q30 | Khani ngingephansi kwemnyaka lelishumi nesiphohlongo beyingangijabulisi indlela bengikhulumisana ngayo nebatali bami esikhatsini lesinengi. | | | |  |  | | | |
| Q31 | Khani ngingephansi kweminyaka lelishumi nesiphohlongo make wami abekalalelana. | | | |  | | | |  |
| Q32 | Khani ngingephansi kweminyaka lelishumi nesiphohlongo babewami abekalalelana | | | |  | | | |  |
| Q33 | Khani ngingephansi kweminyaka lelishumi nesiphohlongo babe wami bekakhombisa kungitsandza | | | |  | | | |  |
| Q34 | Khani ngengephansi kweminyaka lelishumi nesiphohlongo make wami abekhombisa kungitsandza | | | |  | | | |  |
| Q35 | Khani ngingephansi kweminyaka lelishumi nesiphohlongo make wami abekakhulumisana kahle name ngetinkinga | | | |  | | | |  |
| Q36 | Khani ngingephansi kweminyaka lelishumi nesiphohlongo babe wami abekakhulumisana kahle name ngetinkinga | | | |  | | | |  |
| Q37 | Khani ngingephansi kweminyaka lelishumi nesiphohlongo make wami abevama kungitjela imibono nematsemba akhe | | | |  | | | |  |
| Q38 | Khani ngingephansi kweminyaka lelishumi nesiphohlongo babe wami abevama kungitjela imibono nematsemba akhe | | | |  | | | |  |
| Q39 | Khani ngingephansi kweminyaka lelishumi nesiphohlongo make abekakhulumisana nami ngetindzaba tetifo tasecasini | | | |  | | | |  |
| Q40 | Khani ngingephansi kweminyaka lelishumi nesiphohlongo babe wami abekakhulumisana nami ngetindzaba tetifo tasecasini | | | |  | | | |  |
| Q41 | Khani ngingephansi kweminyaka lelishumi nesiphohlongo make wami abekakhulumisana nami ngetindzaba tesifo sembulalave | | | |  | | | |  |
| Q42 | Khani ngingephansi kweminyaka lelishumi nesiphohlongo babe wami abekakhulumisana name ngetindzaba tesifo sembulalave | | | |  | | | |  |

| Q43 | Khani ngengephansi kweminyaka lelishumi nesiphohlongo ngangiva ngikhululekile kubuta make wami ngetindzaba tetifo tasecasini | | | | |  |
| --- | --- | --- | --- | --- | --- | --- |
| Q44 | Khani ngingephansi kweminyaka lelishumi nesiphohlongo ngangiva ngikhululekile kubuta babe wami ngetindzaba tetifo tasecasini | | | | |  |
| **Umlandvo ngekutsi unabangaphi bantfwana**  Lesicephu lesilandzelako sichaza ngemibuto ngetindzaba tekulalana khona sitotfola lwati loluncono ngetindzaba tekutala. Sitsandza kuninika sibindzi kutsi sitogcina konkhe lenisitjela kona kuyimfihlo kulesi sifundvo | | | | | | |
| Q45 | | Bewuhlelile yini kutetfwala ngalesikhatsi lesi? | |  | 1. Yebo 2. Chake | |
| Q46 | | Bewuneminyaka lemingaphi nawucala kulalana nemuntfu? | |  |  | |
| Q47 | | Bekaneminyaka lemingaphi lomuntfu lowalalana naye nawucala kulala? | |  | 1. Bekaneminyaka lelishumi noma ngetulu kunami 2. Bekaneminyaka lelishumi ngephansi kunami | |
| Q48 | | Uke walalana nebantfu labangaphi nje emphilweni yakho? | |  |  | |
| Q49 | | Uke walalana nabangaphi nje bantfu kuletinyanga letilishumi nakubilli letengcile? | |  |  | |
| Q50 | | Yini lokukwenta ucabange kutsi bantfu balalana nebantfu labanengi?  (Cela ukhetse yinye imphendvulo kuleti letisiphohlongo) | | 1. Kuhawukela  2. Bafuna imali  3. Kwesaba kuviswa buhlungu singani sakho lonaso nyalo  4. Uma singani sakho lonaso nyalo singakwenelisi ecasini  5. Ngifuna kuva nje kutsi kunjani  6. Kwedukiswa bangani  7. Kungati ngetindzaba tesifo lesingumubulalave  8. Kulingeta ngekulalana nemuntfu  9. Lokunye, cela ukuchaze………………………………………………………………. | | |
| Q51 | | Wake wahlala yini namasihlalisane loyindvodza? | | 1. Yebo      1. Chake | | |
| Q52 | | Nawucala kulalana nemuntfu, wasebentisa lijazi labomake noma labobabe? | | 1. Yebo, lijazi labobabe 2. Yebo, lijazi labomake 3. Chake | | |
| Q53 | | Bekuyini sizatfu sekutsi usebentise lijazi nawucalako kulalana nemuntfu? | | 1. Singani sami sakhetsa kusebentisa lijazi 2. Bengingafuni kumitsa 3. Bengifuna kutivikela ngingatfoli sifo lesingumubulalave 4. Lokunye, chaza | | |
| Q54 | | Bongalisebentisi ngani lijazi nawucala kulalana nemuntfu?  Nakungenteka ube netimphendvulo letingetulu kwayinye cela ukhetse yinye lobocabanga kutsi imcoka kakhulu | | 1. Bukute 2. Bekudulile 3. Bekungakafaneli 4. Salile singani sami 5. Injabulo iphansi 6. Singani sami sitihlole satikhandza site ligciwane 7. Lokunye, chaza | | |
| Q55 | | Ngesikhatsi ugcina kulalana nemuntfu, nasebentisa lijazi labomake noma labobabe? | | 1. Yebo, lijazi labobabe 2. Yebo, lijazi labomake 3. Chake | | |
| Q56 | | Bekuyini sizatfu usebentise lijazi ngesikhatsi ugcina kulalana nemuntfu? | | 1. Singani sami sakhetsa kusebentisa lijazi 2. Bengingafuni kutetfwala 3. Bengifuna kutivikela kutfola ligciwane lembulalave 4. Lokunye, chaze | | |
| Q57 | | Bekuyini sizatfu sekungalisebentisi lijazi nawugcina kulalana nemuntfu?  Nakungenteka kube netizatfu letengca kusinye khetsa lesilocanga kutsi simcoka kakhulu | | 1. Bekute 2. Bekudule kakhulu 3. Bekungakafaneli 4. Salile singani sami 5. Bekwehle lizinga lebumnandzi 6. Singani sami sihlola sakhandza kutsi site sifo sembulalave 7. Lokunye, chaza | | |
| Q58 | | Wake walalana nemuntfu udzakwe tjwala? | | 1. Yebo 2. Chake | | |
| Q59 | | Wake waphocelelwa kulalana nemuntfu ungafuni? | | 1. Yebo 2. Chake | | |
| **Timphawu temuntfu lolalana naye**  Lesicephu lesi semibuto sikhuluma ngesingani sakhomsekucala lowalalana naso nangalesi lolala naso nyalo. | | | | | | |
| Q60 | | | Bosati simo sengati yesingani sakho nocala kulalana naso kwekucala? | 1. Yebo, bekanalo ligciwane 2. Yebo, bekete ligciwane 3. Bengingasati simo sakhe sengati | | |
| Q61 | | | Uyasati simo sengati yesingani sakho lonaso nyalo? | 1. Yebo, bekete ligciwane 2. Yebo, bekanalo ligciwane 3. Angisati simo sakhe sengati | | |
| **Simo sengati yalabebasebenta kulolucwaningo**  Sitsandza kunatisa ngekutsi sitokugcina kuyimfihlo konkhe lesitokukhuma lana. | | | | | | |
| Q62 | | Sinjani simo sakho sengati? | | 1. Sihlobile 2. Singcolile……Yengca uye kumbuto 66 | | |
| Q63 | | Watfola nini kutsi ingati yakho ingcolile?  (Uma sokhohliwe kutsi nini zama kutsi ucagele kahle ngekubekisa nje) | | Lusuku Inyanga Umyaka | | |
| Q64 | | Uma unalo ligciwane, uke wasitjela singani sakho lolalana naso nyalo? | | 1. Yebo ngisitjelile 2. Chake ngisengakamtjeli 3. Uyati sahlola kanyekanye | | |
| Q65 | | Uma unalo ligciwane sowacalile yini emaphilisi ekulindzindzibilisa? | | 1. Yebo 2. Chake | | |
| Q66 | | Sikhona yini sihlobo sakho sengati lesike safa sabulawa ligciwane lembulalave? | | 1. Yebo 2. Chake | | |
| Q67 | | Uma ute leligciwane lembulalave ucabanga kutsi ematfuba akho ekulitfola mangaphi? | | Kuletinyanga letitako   1. Asetulu 2. Aphansi | | |
| **Lwati ngeligciwane lembulalave** | | | | | | |
| Q68 | | Bangawehlisa yini bantfu ematfuba ekutfola ligciwane lesifo sembulalave ngekuba nesingani sinye lesite letinye tingani? | | 1. Yebo 2. Chake 3. Angati | | |
| Q69 | | Bantfu bangalitfola yini leligciwane ngekusutelwa yimbuzulwane? | | 1. Yebo 2. Chake 3. Angati | | |
| Q70 | | Ngabe bantfu bangawehlisa yini ematfuba ekutfola ligciwane lembulalave nabangasentisa lijazi njalo nje nabalalanako? | | 1. Yebo 2. Chake 3. Angati | | |
| Q71 | | Bantfu bangewehlisa yini ematfuba ekutfola ligciwane lembulalave ngekutsi bangalali sanhlobo? | | 1. Yebo 2. Chake 3. Angati | | |
| Q72 | | Bantfu bangilitfola yini ligciwane lembulalave ngekuloywa noma ngamadimoni? | | 1. Yebo 2. Chake 3. Angati | | |
| Q73 | | Bantfu bangalitfola yini leligciwane lembulalave etilondzeni letivulekile temuntfu lonaleligciwane lembulalave? | | 1. Yebo 2. Chake 3. Angati | | |
| Q74 | | Kungenteka yini kutsi umuntfu lobukeka aphilile abe nalo yini leligciwane lembulalave? | | 1. Yebo 2. Chake 3. Angati | | |
| Q75 | | Do you know of a place where people can go to get tested for the AIDS virus?  Kukhona yini indzawo loyatiko bantfu labangaya kotihlola khona ligciwane? | | 1. Yebo 2. Chake 3. Angati | | |
| Q76 | | Kukhona yini imibono longaciphisa ngayo bantfu labasebenta ngetindzaba tekuvikela sifo sembulalave kubomake labatetfwele? | | | | |
| ***Sewufike ekugcineni kwalelihlolo, cela ukhululeke kusibuta imibuto lengahle ikuvelele. Siyabonga kakhulu kungenela lelihlolo!*** | | | | | | |
|  |  | | |  | | |

**Study Questionnaire**

| **Questionnaire for pregnant women**  **Faculty name** | | | | | | | |  |  |
| --- | --- | --- | --- | --- | --- | --- | --- | --- | --- |
| **#** | | **QUESTION** | **RESPONSE** | | | | |  |  |
| **Socio Demographic Information** | | | | | | | |  |  |
| Q1 | | How old are you? |  | | | | |  |  |
| Q2 | | What is your current marital status? | 1. Single (Never married and not living with a partner 2. Married 3. Living with a partner 4. Separated (currently not living together but not divorced 5. Divorced 6. Widowed | | | | |  |  |
| Q2b | | Beside yourself, does your husband have other wives/wife?  Only asked to those who are married | 1. Yes  2. No | | | | |  |  |
| Q3 | | What is your current highest level of education?  Please indicate the level you completed e.g if you ended in form 4 then your highest level of education is secondary. | 1. Less than primary school 2. Primary level 3. Secondary level 4. High School level 5. Tertiary level | | | | |  |  |
| Q4 | | While you were a school going child have you ever stayed at a boarding school (hostel)? | 1. Yes 2. No | | | | |  |  |
| Q5 | | Did you ever drop out of school? | 1. Yes 2. No……………………………………..Skip to Q7 | | | | |  |  |
| Q6 | | If you dropped out of school please indicate your reason for dropping out?  In case there are **more than one** reasons please choose the one you think was the most important reason | 1. Fell pregnant 2. Found a boyfriend 3. Got married 4. Did not have finacial support 5. Did not do well at school 6. Other please specify…………………………………………………………………….. | | | | |  |  |
| Q7 | | What is your current employment status? | 1. Employed 2. Not employed 3. Student 4. Self employed | | | | |  |  |
| Q8 | | Did the family you grew up in have the following household items?  (in case you have changed households please make the place you spend the most time your reference point)  (In case your family’s status changed as you grew up, please make the status that lasted the longest your reference point) | Yes No   \| 1. Electricity \| ① \| ⓪ \|  \| \| --- \| --- \| --- \| --- \| \| 1. Radio \| ① \| ⓪ \|  \| \| 1. Television \| ① \| ⓪ \|  \| \| 1. Mobile phone \| ① \| ⓪ \|  \| \| 1. Non-mobile phone \| ① \| ⓪ \|  \| \| 1. Refrigerator \| ① \| ⓪ \|  \| \| 1. Stove \| ① \| ⓪ \|  \| \| 1. Watch/clock \| ① \| ⓪ \|  \| | | | | |  |  |
| Q9 | | What type of stove did the household you grew up in use for cooking?  (in case you have changed households please make the place you spend the most time your reference point)  (In case your family’s status changed as you grew up, please make the status that lasted the longest your reference point) | Yes No   \| 1. Electric stove \| ① \| ⓪ \|  \| \| --- \| --- \| --- \| --- \| \| 1. Charcoal stove \| ① \| ⓪ \|  \| \| 1. Wood-based stove \| ① \| ⓪ \|  \| \| 1. Gas stove \| ① \| ⓪ \|  \| \| 1. Paraffin stove \| ① \| ⓪ \|  \| \| 1. Coal stove \| ① \| ⓪ \|  \| | | | | |  |  |
| Q10 | | Did any household member own any of the following?  (in case you have changed households please make the place you spend the most time your reference point)  (In case your family’s status changed as you grew up, please make the status that lasted the longest your reference point) | Yes No   \| 1. Bicycle \| ① \| ⓪ \|  \| \| --- \| --- \| --- \| --- \| \| 1. A motorcyle or motor scooter \| ① \| ⓪ \|  \| \| 1. An animal-drown cart \| ① \| ⓪ \|  \| \| 1. A car or a truck \| ① \| ⓪ \|  \| \| 1. A tractor \| ① \| ⓪ \|  \| | | | | |  |  |
|  |  |  |  | | | | |  |  |
| Q11 | | What is your (personal) current total monthly income from all sources?  If no income please select (less than E249) | 1. Less than E249 2. More than E249 but less than E1749 3. More than E1749 but less than E3000 4. More than 3000 | | | | |  |  |
| Q12 | | **When you were less than 18 years old** how often can you say you attended religious services? | 1. Everyday 2. At least once a week 3. At least once a month 4. At least once a year 5. Less than once a year 6. Never | | | | |  |  |
| **Family characteristics**     \| ① \| ② \| ③ \| ④ \| ⑤ \| \| --- \| --- \| --- \| --- \| --- \| \| **Strongly**  **Disagree** \| **Generally disagree** \| **Undecided** \| **Generally Agree** \| **Strongly**  **Agree** \| | | | | | | | |  |  |
| The following section is related to your family history and how you and your family members related to each other when you were less than 18 years old. Please indicate your level of agremment or disagreement with the statements below by choosing one of the five possible responses for each statement. | | | | | | | |  |  |
| Q13 | | While I was less than 18 years old family members got along better with people outside our family than inside. | |  | | | |  |  |
| Q14 | | While I was less than 18 years old we spent enough time with each other (each other refers to other members of your family) | |  | | | |  |  |
| Q15 | | While I was less than 18 years old we never seemed to get organized in my family | |  | | | |  |  |
| Q16 | | While I was less than 18 years old family members felt very close to each other | |  | | | |  |  |
| Q17 | | While I was less than 18 years old family members seemed to avoid each other | |  | | | |  |  |
| Q18 | | While I was less than 18 years old family members were supportive of each other during difficult times | |  | | | |  |  |
| Q19 | | While I was less than 18 years old it was unclear who was responsible for things such as chores and activities in my family | |  | | | |  |  |
| Q20 | | While I was less than 18 years old my family rarely did things together | |  | | | |  |  |
| Q21 | | One of my parents had other sexual partner(s) (extra marital relationships) | | 1. Yes 2. No 3. I don’t know | | | |  |  |
| Q22 | | My father had more than one wife? | | 1. Yes 2. No 3. I don’t know | | | |  |  |
| **Parental Monitoring**   \| ① \| ② \| ③ \| ④ \| ⑤ \| \| --- \| --- \| --- \| --- \| --- \| \| **Strongly**  **Disagree** \| **Generally disagree** \| **Undecided** \| **Generally Agree** \| **Strongly**  **Agree** \| | | | | | | | |  |  |
| The following section is related to parental monitoring when you were less than 18 years old. If you did not live most of her childhood life with your biological family, please make reference to your guardian family. Please indicate your level of agremment or disagreement with the statements below by choosing one of the five possible responses for each statement. | | | | | | | |  |  |
| Q23 | | When I was less than 18 years old there were strict consequences for breaking the rules in my family | |  | | |  |  |  |
| Q24 | | While I was less than 18 years old, when I went out at night, my parent(s) knew where I was | |  | | |  |  |  |
| Q25 | | While I was less than 18 I was required to always tell my parent(s) who I was going to be with before I went out  For example if you went with friends | |  | | |  |  |  |
| Q26 | | While I was less than 18 years old there were rules for almost every situation in my family | |  | | |  |  |  |
| Q27 | | While I was less than 18 years old once my parents made a decision it was very difficult to modify that decision | |  | | |  |  |  |
| Q28 | | When I was less than 18 years old my parents always knew when I come back 1 hour late from school/church/ or activities outside the home | |  | | |  |  |  |
| **Parent child communication**   \| ① \| ② \| ③ \| ④ \| ⑤ \| \| --- \| --- \| --- \| --- \| --- \| \| **Strongly**  **Disagree** \| **Generally disagree** \| **Undecided** \| **Generally Agree** \| **Strongly**  **Agree** \| | | | | | | | | |  |
| The following section is related to parent-child communication when you were less than 18 years old. If you did not live most of her childhood life with your biological parents, please make reference to the male guardian for father and female guardian for mother. Please indicate your level of agremment or disagreement with the statements below by choosing one of the five possible responses for each statement. | | | | | | | | |  |
| Q29 | While I was less than 18 years old I was satisfied with how I communicated with my parents **most of the time** | | | |  |  | | | |
| Q30 | While I was less than 18 years old I was not satisfied with the way my parents communicated with me **most of the time** | | | |  |  | | | |
| Q31 | While I was less than 18 years old my mother was a good listener | | | |  | | | |  |
| Q32 | While I was less than 18 years old my father was a good listener | | | |  | | | |  |
| Q33 | While I was less than 18 years old my father expressed affection to me | | | |  | | | |  |
| Q34 | While I was less than 18 years old my mother expressed affection to me | | | |  | | | |  |
| Q35 | While I was less than 18 years old my mother calmly discussed problems with me | | | |  | | | |  |
| Q36 | While I was less than 18 years old my father calmly discussed problems with me | | | |  | | | |  |
| Q37 | While I was less than 18 years old my mother discussed her ideas and beliefs with me | | | |  | | | |  |
| Q38 | While I was less than 18 years old my father discussed his ideas and beliefs with me | | | |  | | | |  |
| Q39 | While I was less than 18 years old my mother discussed sexual health issues with me | | | |  | | | |  |
| Q40 | While I was less than 18 years old my father discussed sexual health issues with me | | | |  | | | |  |
| Q41 | When I was less than 18 years old my mother discussed related HIV issues with me | | | |  | | | |  |
| Q42 | When I was less than 18 years old my father discussed related HIV issues with me | | | |  | | | |  |

| Q43 | While I was less than 18 years old I felt comfortable to ask my mother questions about sexual health | | | | |  |
| --- | --- | --- | --- | --- | --- | --- |
| Q44 | While I was less than 18 years old I felt comfortable to ask my father questions about sexual health | | | | |  |
| **Sexual reproductive history**  The following section relates to questions about sexual activity in order to gain a better understanding of some reproductive health issues. Once more we would like to assure you of the confidentially and anonymous nature of this survey. | | | | | | |
| Q45 | | Were you planning to have this particular pregnancy at this time? | |  | 1. Yes 2. No | |
| Q46 | | How old were you when you had your first sexual intercourse? | |  |  | |
| Q47 | | How old was this person you had sexual intercourse with for the first time? | |  | 1. Ten or more years older than me 2. Less than ten years older than me | |
| Q48 | | How many sexual partners have you ever had? | |  |  | |
| Q49 | | How many sexual partners have you had in the past 12 months? | |  |  | |
| Q50 | | Why do you think people have multiple sexual partners?  (Please pick one of the 8 reasons) | | 1. Lust  2. Financial benefit  3. Fear of being disappointed by current sexual partner  4. Sexually unsatisfied with current partner  5. Looking for adventure  6. Peer pressure  7. Lack of knowledge of risks of HIV  8. Get tempted to have sex  9. Other, please specify………………………………………………………………. | | |
| Q51 | | Have you ever lived with a man as if married? | | 1. Yes      1. No | | |
| Q52 | | **The first time** you had sexual intercourse, was a male condom or female condom used? | | 1. Yes, Male condom 2. Yes, Female condom 3. No | | |
| Q53 | | What was the main reason you used a condom the first time you had sexual intercourse? | | 1. Partner decided to use a condom 2. I did not want to get pregnant 3. I wanted to protect myself from HIV 4. Other specify | | |
| Q54 | | What was the main reason you did not use a condom the first time you had sexual intercourse?  In case there are **more than one** reasons please choose the one you think was the most important reason | | 1. It was not available 2. It was too expensive 3. It was not necessary 4. Partner refused 5. Reduced pleasure 6. My patner tested HIV negatieve 7. Other specify | | |
| Q55 | | **The last time** you had sexual intercourse, was a male condom or female condom used? | | 1. Yes, Male condom 2. Yes, Female condom 3. No | | |
| Q56 | | What was the main reason you used a condom the last time you had sexual intercourse? | | 1. Partner decided to use a condom 2. I did not want to get pregnant 3. I wanted to protect myself from HIV 4. Other specify | | |
| Q57 | | What was the main reason you did not use a condom the last time you had sexual intercourse?  In case there are **more than one** reasons please choose the one you think was the most important reason | | 1. It was not available 2. It was too expensive 3. It was not necessary 4. Partner refused 5. Reduced pleasure 6. My patner tested HIV negative 7. Other specify | | |
| Q58 | | Have you ever had sexual intercourse while under the influence of alcohol? | | 1. Yes 2. No | | |
| Q59 | | Have you ever been forced to have sexual intercourse against your will? | | 1. Yes 2. No | | |
| **Sexual partner’s charecteristics**  This section of the questionnaire relates to information about your first sexual partner and your current sexual partner. | | | | | | |
| Q60 | | | Did you know your **first** sexual partner’s HIV status at the time you had sex with him? | 1. Yes, HIV Negative 2. Yes, HIV Positive 3. No, I didn’t know his HIV status | | |
| Q61 | | | Do you know your **current** sexual partner’s HIV status? | 1. Yes, HIV Negative 2. Yes, HIV Positive 3. No I don’t know his status | | |
| **Participants HIV status**  Once more we would like to assure you of the confidentially and anonymous nature of this survey. | | | | | | |
| Q62 | | What is your HIV status? | | 1. Positive 2. Negative ……Skip to Q66 | | |
| Q63 | | When did you find out for the first time you were HIV Positive?  (If you do not remember the exact date please try to give your best estimate) | | DD MM YYYY | | |
| Q64 | | If HIV positive, have you disclosed to your current sexual partner? | | 1. Yes I have disclosed to him 2. No I have not yet dislosed to him 3. He already knows, we tested together | | |
| Q65 | | If HIV positive are you currently on antiretroviral treatment | | 1. Yes 2. No | | |
| Q66 | | Has any of your family members died because of HIV/AIDS? | | 1. Yes 2. No | | |
| Q67 | | If HIV negative what do you think are your chances of getting HIV infected? | | In the next months   1. High 2. Low | | |
| **HIV Knowledge** | | | | | | |
| Q68 | | Can people reduce their chances of getting the AIDS virus by having just one sex partner who is not infected and who has no other partners? | | 1. Yes 2. No 3. I don’t know | | |
| Q69 | | Can people get the AIDS virus from mosquito bites? | | 1. Yes 2. No 3. I don’t know | | |
| Q70 | | Can people reduce their chances of getting the AIDS virus by using a condom every time they have sex? | | 1. Yes 2. No 3. I don’t know | | |
| Q71 | | Can people reduce their chance of getting the AIDS virus by abstaining from sexual intercourse? | | 1. Yes 2. No 3. I don’t know | | |
| Q72 | | Can people get the AIDS virus because of witchcraft or other supernatural means? | | 1. Yes 2. No 3. I don’t know | | |
| Q73 | | Can people get the AIDS virus from open wounds or sores of an infected person? | | 1. Yes 2. No 3. I don’t know | | |
| Q74 | | Is it possible for a healthy-looking person to have the AIDS virus? | | 1. Yes 2. No 3. I don’t know | | |
| Q75 | | Do you know of a place where people can go to get tested for the AIDS virus? | | 1. Yes 2. No 3. I don’t know | | |
| Q76 | | Do you have any advice you would like to give to HIV prevention officials on how they can improve services for pregnant women? | | | | |
| ***You have now reached the end of the survey, please feel fee to ask us any question you might have.***  ***Thank you very much for participanting in this survey!*** | | | | | | |
|  |  | | |  | | |
